# A contribution towards checklist of fungus gnats (Diptera, Diadocidiidae, Ditomyiidae, Bolitophilidae, Keroplatidae, Mycetophilidae) in Georgia, Transcaucasia

**DOI:** 10.3897/zookeys.1026.63749

**Published:** 2021-03-26

**Authors:** Olavi Kurina

**Affiliations:** 1 Institute of Agricultural and Environmental Sciences, Estonian University of Life Sciences, Kreutzwaldi st 5 D, 51006 Tartu, Estonia Estonian University of Life Sciences Tartu Estonia

**Keywords:** Fungus gnats, Georgia, new species, Sciaroidea, species diversity, taxonomy, Transcaucasia

## Abstract

The fungus gnats of Georgia are studied based on 2682 specimens collected from 57 localities during 2011–2019. Altogether, 245 species are recorded including four species of Bolitophilidae, three species of Diadocidiidae, two species of Ditomyiidae, 34 species of Keroplatidae and 202 species of Mycetophilidae. 230 and 188 species are recorded from Georgia and the whole of Transcaucasia for the first time, respectively. Three new species – *Sciophila georgei* sp. nov., *Leia katae* sp. nov. and *Anatella metae* sp. nov. – are described including detailed illustrations of the male terminalia. Photographs are provided for an additional 38 species to highlight a variability of their general facies. Combined with earlier published data, the number of fungus gnat species in Georgia is set at 246. The estimated diversity of fungus gnats in Georgia is calculated using non-parametric methods and discussed with respect to other Western Palaearctic regions.

## Introduction

The last decades can be characterized by an upturn of systematics, taxonomy and biodiversity studies (e.g. Padial et al. 2010; Kõljalg et al. 2020; Wheeler 2020). That is also true in the case of the insects order Diptera (e.g. Wiegmann et al. 2011; Kirk-Spriggs and Sinclair 2017; Borkent et al. 2018) including the superfamily Sciaroidea (e.g. Kjaerandsen et al. 2007; Borkent and Wheeler 2012; Ševčík et al. 2013; Fitzgerald and Kerr 2014). Seven families and a *insertae sedis* group are included in Sciaroidea (Ševčík et al. 2016; Mantič et al. 2020), whereas five of them, viz. Diadocidiidae, Ditomyiidae, Bolitophilidae, Keroplatidae and Mycetophilidae are conjoined under a common name ‘fungus gnats’. Today, more than 5,500 species of fungus gnats are known globally (Evenhuis and Pape 2021; Fungus Gnats Online Authors 2021), however, their actual diversity is insufficiently known, especially in tropical regions of the world. As expected, the group is best studied in Europe with about 1,200 named species (Chandler 2013) yielded by more than 200 years of studies pioneered by the “father” of dipterology J.W. Meigen (e.g. Meigen 1804, 1818). Nevertheless, even in Europe, new species are described annually and e.g. in Nordic countries nearly 120 new species are waiting to be described (Kjærandsen and Søli 2020). While fungus gnats are mostly forest dwellers preferring shady and humid habitats, some species are also recorded from more open landscapes (Falk and Chandler 2005). They are small to medium size nematocerous flies with a humpbacked habitus, prominent coxae and hyaline or patterned wings (see e.g. Figs 8, 9). The trophic strategy of fungus gnats is diverse: the majority of the known associations are those with fungal fruiting bodies or mycelium-penetrated forest litter including decaying wood but several species develop in other terrestrial habitats and/or can also be sporophagous or predators in the larval stage (e.g. Matile 1997; Ševčík 2010; Jakovlev 2012; Põldmaa et al. 2016; Mantič et al. 2020).

Transcaucasia, the area southwards from the Greater Caucasus Mountains that includes the countries of Georgia, Azerbaijan and Armenia, is considered one of the biodiversity hotspots of the world, with a remarkable number of endemic species (Myers et al. 2000). However, limited attention has been paid to the biodiversity research in the area so far (Mumladze et al. 2020) and most organism groups, including Diptera and fungus gnats in particular, are rather superficially studied. There are 33 species of fungus gnats recorded from Azerbaijan (Zaitzev 1994, 2003; Zaitzev and Ševčík 2003) and seven species from Armenia (Joost and Plassmann 1985, Zaitzev 1994). From Georgia, only one species was known (Zaitzev 1994) prior to Kurina and Jürgenstein (2013) who described two new *Orfelia* Costa (Keroplatidae) species from Marelisi, NW of Borjomi. Later on, Jürgenstein et al. (2015), Kurina et al. (2015), Kurina (2018), Thormann et al. (2019) and Ševčík et al. (2020) provided data on another twelve species and the number of fungus gnat species from Georgia is currently set at 15. Furthermore, an additional 24 fungus gnat species have been listed to occur in Transcaucasia but without a specified region (Zaitzev 1994, 2003). Concerning neighbouring areas, 91 species of fungus gnats are recorded from the northern slopes of the Great Caucasus ridge, most of them from the surroundings of Mt Elbrus (Joost and Plassmann 1976, 1979, 1985, 1992, Plassmann 1976).

During the last decade, a considerable amount of fungus gnat material from Georgia has accumulated in the author’s possession. The aim of the current contribution is to provide results of the study based on that material along with summarising all available published information on Georgian fungus gnats.

## Material and methods

**Figure 1.**
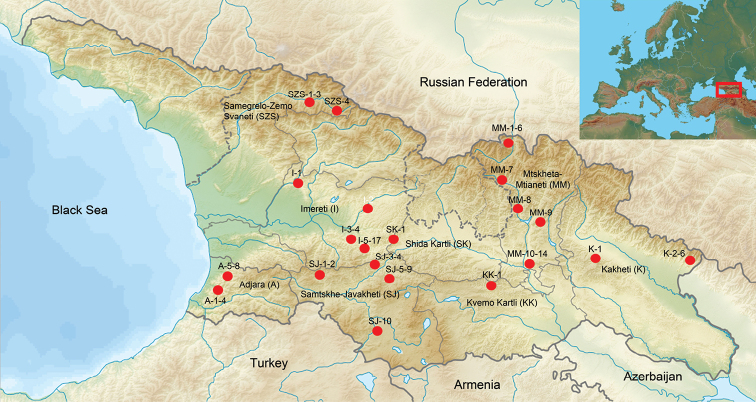
Collecting localities in Georgia. The codes are those used in Table 1.

**Figure 2.**
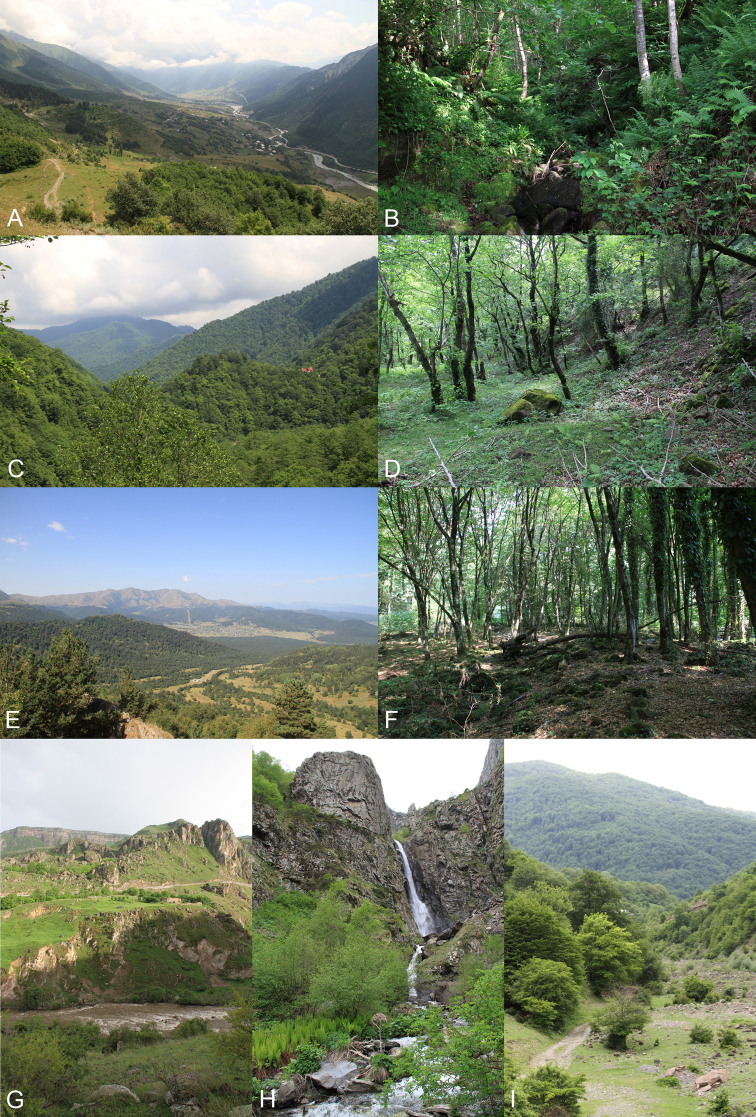
A gallery of collecting localities in Georgia. For codes see Table 1**A** Mestia (SZS-1) **B** Mtirala NP (A-1) **C** Kintrishi NP (A-5) **D** Marelisi (I-12) **E** Bakurjani (SJ-6) **F** Lagotekhi (K-3) **G** Vardzia (SJ-10) **H** Gvelethi near Stepantsminda (MM-5) **I** Dgnali (MM-8).

The material was collected from 2011 to 2019 using different methods in the course of 61 collecting events from 57 localities in Georgia (Table 1, Fig. 1). The majority of the material was collected sweeping during three expeditions by the author in May of 2012 and 2013 and August-September 2014. Additional material from Malaise trap samples is included from the provinces Samegrelo-Zemo-Svanethi, Imereti and Kaheti; a sporadic material as a by-product of light trap collecting is also included (Table 1). The collecting localities (see Fig. 2 for examples) varied from more open landscape in Vardzia (Table 1: SJ-10) to highly forested mountain areas in Kintrishi (Table 1: A-5–8), Mtirala (Table 1: A-1–4) and Borjomi-Kharagauli (Table 1: I-5–17) National Parks, and subalpine areas in the surroundings of Stepantsminda (Table 1: MM-1–6), Bakuriani (Table 1: SJ-6–7) and Ushguli (Table 1: SZS-2–3).

**Table 1.** Collecting data of fungus gnats in Georgia arranged by administrative regions of the country. Codes for the collecting events are used on the Figure 1 and within the list of species. The asterisk (*) indicates collecting with a Malaise trap for which the exact collecting dates are provided in the text.

Region	Locality	Coordinates	Altitute (m)	Collecting date(s)	Collecting method	Collector	Code
Samegrelo-Zemo Svanethi	Mestia	43°02.97'N, 42°44.72'E	1500	28.vii.2017	light trap	O. Kurina	SZS-1
	Chvabiani	43°02.47'N, 42°51.03'E	1630	29.vii.2017	light trap	O. Kurina	SZS-2
	S of Lakhushdi, meadow	42°59.93'N, 42°39.02'E	1270	13–14.vi.2019	Malaise trap	X. Mengual	SZS-3
	near Ushguli, path to glacier	42°56.62'N, 43°03.23'E	2220	15–17.vi.2019	Malaise trap	X. Mengual	SZS-4
Adjara	Mtirala NP, visitor centre	41°40.65'N, 41°51.30'E	240	19.v.2013	sweeping	O. Kurina	A-1
	Mtirala NP, visitor centre	41°40.65'N, 41°51.33'E	230	19.v.2013	at light	O. Kurina	A-2
	Mtirala NP, visitor centre	41°40.35'N, 41°52.53'E	270	20.v.2013	sweeping	O. Kurina	A-3
	Mtirala NP, visitor centre	41°40.91'N, 41°50.70'E	220	20.v.2013	at light	O. Kurina	A-4
	Kintrishi NP	41°45.76'N, 41°58.67'E	320	21.v.2013	sweeping	O. Kurina	A-5
	Kintrishi NP	41°45.76'N, 41°58.67'E	320	21.v.2013	at light	O. Kurina	A-6
	Kintrishi NP	41°45.20'N, 41°58.63'E	450	22.v.2013	sweeping	O. Kurina	A-7
	Kintrishi NP	41°46.40'N, 41°58.08'E	460	22.v.2013	sweeping	O. Kurina	A-8
Imereti	Chiatura	42°17.00'N, 43°17.00'E	480	17.v.2011	light trap	U. Jürivete	I-1
	Tshunkuri	42°24.00'N, 42°35.00'E	230	20.v.2011	light trap	U. Jürivete	I-2
	Patara Vardzia, W of Kharagauli	42°0.55'N, 43°04.62'E	740	v-x.2013*	Malaise trap	O. Kurina	I-3
	Patara Vardzia, W of Kharagauli	42°01.32'N, 43°11.10'E	370	18.v.2013	sweeping	O. Kurina	I-4
	Marelisi	41°57.07'N, 43°17.02'E	430	18.v.2012	sweeping	O. Kurina	I-5
	Marelisi	41°57.93'N, 43°17.35'E	410	19.v.2012	sweeping	O. Kurina	I-6
	Marelisi, on road to railway station	41°58.02'N, 43°17.35'E	440	19.v.2012	at light	O. Kurina	I-7
	Marelisi	41°57.00'N, 43°17.00'E	460	20.v.2012	indoors	O. Kurina	I-8
	Marelisi, on path to hill	41°56.38'N, 43°16.62'E	690	20.v.2012	sweeping	O. Kurina	I-9
	Marelisi	41°56.80'N, 43°17.05'E	450	20.v.2012	sweeping	O. Kurina	I-10
	Marelisi	41°58.02'N, 43°17.38'E	400	17.v.2013	sweeping	O. Kurina	I-11
	Marelisi	41°56.28'N, 43°16.98'E	460	17.v.2013	sweeping	O. Kurina	I-12
	Marelisi	42°56.46'N, 43°17.05'E	460	18.v.2013	al light	O. Kurina	I-13
	Marelisi	41°56.28'N, 43°16.98'E	460	29.viii.2014	sweeping	O. Kurina	I-14
	Marelisi	41°56.38'N, 43°16.47'E	760	30.viii.2014	sweeping	O. Kurina	I-15
	Marelisi, close to railway station	41°58.23'N, 43°18.65'E	400	20.v.2012	al light	O. Kurina	I-16
	Marelisi, close to railway station	41°58.14'N, 43°18.63'E	410	23.v.2013	sweeping	O. Kurina	I-17
Shida-Kartli	W of Surami	42°01.57'N, 43°29.88'E	940	18.v.2012	sweeping	O. Kurina	SK-1
Samtskhe-Javakheti	road from Abastumani to Saime, near river	41°46.63'N, 42°50.23'E	1370	10–11.vi.2019	Malaise trap	X. Mengual	SJ-1
	road from Abastumani to Saime	41°47.82'N, 42°50.63'E	1730	10–11.vi.2019	Malaise trap	X. Mengual	SJ-2
	Borjomi 3 km--W, Likani	41°50.15'N, 43°19.95'E	940	21.v.2012	sweeping	O. Kurina	SJ-3
	Borjomi 3.5 km--W, Likani	41°50.11'N, 43°19.92'E	950	31.viii.2014	sweeping	O. Kurina	SJ-4
	Bakuriani 2 km--NW	41°45.77'N, 43°30.28'E	1630	31.viii.2014	sweeping	O. Kurina	SJ-5
	Bakuriani 5 km--S, road from Bakuriani to Tabatskuri	41°42.33'N, 43°30.13'E	2120	1.ix.2014	sweeping	O. Kurina	SJ-6
	Bakuriani 3 km--SW, road from Bakuriani to Tabatskuri	41°43.33'N, 43°29.87'E	1870	1.ix.2014	sweeping	O. Kurina	SJ-7
	Bakuriani 1 km--SW, road from Bakuriani to Tabatskuri	41°44.22'N, 43°30.75'E	1740	1.ix.2014	sweeping	O. Kurina	SJ-8
	Bakuriani 2 km--NW	41°45.77'N, 43°30.28'E	1630	1.ix.2014	sweeping	O. Kurina	SJ-9
	Vardzia, near Tirebi guesthouse	41°24.17'N, 43°19.23'E	1260	22.v.2012	at light	O. Kurina	SJ-10
Mtskhetha-Mthianethi	Stephantsminda	42°39.28'N, 44°39.28'E	1870	15.v.2012	at light	O. Kurina	MM-1
	Stephantsminda, road to Gegriti Trinity Church	42°39.77'N, 44°37.50'E	1980	16.v.2012	sweeping	O. Kurina	MM-2
	Gvelethi NW of Stepantsminda	42°42.28'N, 44°37.27'E	1640	16.v.2012	at light	O. Kurina	MM-3
	Gvelethi NW of Stepantsminda, surroundings of lake	42°43.37'N, 44°37.12'E	1520	17.v.2012	sweeping	O. Kurina	MM-4
	Gvelethi NW of Stepantsminda, surroundings of waterfall	42°42.23'N, 44°37.20'E	1570	17.v.2012	sweeping	O. Kurina	MM-5
	Stephantsminda, road to Gegriti Trinity Church	42°40.02'N, 44°37.15'E	2090	17.v.2012	at light	O. Kurina	MM-6
	Gudauri	42°26.23'N, 44°29.95'E	1780	8.vii.2019	light trap	A. Selin	MM-7
	Dgnali	42°13.43'N, 44°40.02'E	910	15.v.2012	sweeping	O. Kurina	MM-8
Mtskhetha-Mthianethi	Zaridzeebi	42°42.08'N, 44°54.00'E	870	22.v.2011	light trap	U. Jürivete	MM-9
	Saguramo	41°54.00'N, 44°46.00'E	600	16.v.2011	light trap	U. Jürivete	MM-10
	Saguramo	41°53.07'N, 44°46.78'E	920	15.v.2012	sweeping	O. Kurina	MM-11
	Saguramo	41°53.07'N, 44°46.78'E	920	15.v.2013	sweeping	O. Kurina	MM-12
	Saguramo	41°53.07'N, 44°46.78'E	920	28.viii.2014	sweeping	O. Kurina	MM-13
	Saguramo	41°53.13'N, 44°46.73'E	890	4.ix.2014	sweeping	O. Kurina	MM-14
Kvemo Kartli	Manglisi 6 km–S	41°39.89'N, 44°23.10'E	1190	23.v.2012	sweeping	O. Kurina	KK-1
Kakheti	Dzveli Shuamta W of Telavi	41°54.60'N, 45°24.33'E	1000	2.ix.2014	sweeping	O. Kurina	K-1
	Gurgeniani, W of Lagotekhi	41°52.67'N, 46°14.55'E	630	3.ix.2014	sweeping	O. Kurina	K-2
	Matsimi near Lagotekhi	41°48.55'N, 46°18.73'E	440	3.ix.2014	at light	O. Kurina	K-3
	Lagodekhi NR, near administration building	41°50.50'N, 46°16.98'E	560	28.v–9.vi.2011	Malaise trap	G. Japoshvili	K-4
	Lagodekhi NR, Matsimi river gorge	41°47.75'N, 46°17.12'E	350	17–27.v.2011	Malaise trap	G. Japoshvili	K-5
	Lagodekhi NR (Malaise trap #3)	unavailable		15–25.vi.2014	Malaise trap	G. Japoshvili	K-6

The vast majority of the material was initially preserved in 70% ethyl alcohol where most of it is also stored after determination. Every species per locality is arranged in a separate glass vial equipped with collecting and determination labels. Some specimens were double pinned directly after collecting, whereas part of the initially alcohol-preserved specimens were mounted using the method described by Vockeroth (1966) and double pinned thereafter. The majority of the material was determined directly in alcohol as that also allowed observation of the terminalia. However, in a number of cases a more detailed study of male terminalia proved to be unavoidable. For that, terminalia were detached and treated with about 10% warm potassium hydroxide followed by neutralization with acetic acid and washing with distilled water. Terminalia were studied in glycerine and stored as glycerine preparations in small plastic vials attached to the rest of the specimen (see also Kurina 2008a).

Illustrations of the terminalia were prepared using a U-DA drawing tube attached to a compound microscope Olympus CX31. The digital images of the general habitus and terminalia were combined using the software LAS V.4.1.0. from multiple gradually focused images taken by a Leica DFC 450 camera attached to a Leica 205C stereomicroscope (see also Jürgenstein et al. 2015). Adobe Photoshop CS5 was used for editing the figures and compiling the plates. The morphological terminology follows Søli (1997, 2017) and that of the male terminalia is explained in Figs 4–7. The estimated species richness according to different non-parametric methods (Fig. 14) is calculated using the software EstimateS, Version 9.1.0. (Colwell 2013).

The material is deposited in the following collections:

**IUTG** Ilia State University, Tbilisi, Georgia;

**IZBE**Institute of Agricultural and Environmental Sciences, Estonian University of Life Sciences (former Institute of Zoology and Botany), Tartu, Estonia;

**ZFMK**Zoological Research Museum Alexander Koenig, Bonn, Germany.

The majority of the studied specimens are deposited in IZBE which is not repeated in the species list for every specimen. However, the material collected by X. Mengual (Bonn, Germany) in 2019 is divided between three institutions and the depository is specified in listed material sections below.

## Results

Altogether, 2682 studied specimens were identified to 245 different species, viz. four species of Bolitophilidae, three species of Diadocidiidae, two species of Ditomyiidae, 34 species of Keroplatidae and 202 species of Mycetophilidae including three species described as new to science. One additional species of Keroplatidae was included from the literature data (Zaitzev 1994). Moreover, six additional putatively new Mycetophilidae species were recorded, all represented by singletons, some of them of poor quality. Description of these species is deferred pending additional material to be collected. These six species are not included in the species list but are considered in the species richness calculations and distribution analysis (see Discussion). 230 and 188 species are recorded from Georgia and the whole Transcaucasia for the first time, respectively. In the species list, all available literature sources are cited for the species recorded earlier in Georgia and/or in Transcaucasia generally. The studied material is listed, using abbreviations of collecting events provided in Table 1, followed by total number of studied specimens. Distribution in Georgia is given by administrative provinces and the general distribution by zoogeographical regions or subregions. The latter is provided according to Chandler (2013) and subsequent published information available. Some remarks on distribution and/or taxonomy are included for species of special interest. To illustrate the diverse habitus of recorded fungus gnat species a gallery of photographs is provided (Figs 8, 9, 11, 12). In the list of species, the classification follows Fungus Gnats Online (http://www.sciaroidea.info/) except in two cases. Firsty, the subfamily Platyurinae of Keroplatidae is used according to Mantič et al. (2020). Secondy, *Brachycampta* Winnertz, 1863 is reinstated to the generic status from a subgenus of *Allodia* Winnertz, 1863 in accordance with a thorough study by Magnussen (2020); this opinion is also implemented in the recent checklist of fungus gnats of Norway by Kjærandsen and Søli (2020).

### The new species

#### Sciophila georgei sp. nov.

Taxon classificationAnimaliaDipteraMycetophilidae

B92821CC-8394-5DDE-8E2F-3199603BAFBC

http://zoobank.org/D9E0ED72-E487-480C-A89F-4E6DDD98C406

Figs 3A , 4A–G

##### Type material.

***Holotype*.** Male, Georgia, Kakheti, Lagotekhi NR, Matsimi river gorge, 41°47.75'N, 46°17.12'E, 350 m a.s.l., 17–27.v.2011, Malaise trap, leg. G. Japoshvili [see Table 1: K-5] (mounted from alcohol, IZBE). ***Paratype*.** Male, same as holotype (mounted from alcohol, IZBE).

##### Diagnosis.

*Sciophila georgei* sp. nov. can be distinguished by combination of the characters of the male terminalia as follows: lateral branch of gonostylus ventrally with two apical spine-like setae, small internal branch of gonostylus with one spine-like seta, tergite 9 large with medially rising apical margin that bears two large and simple setae, parameres straight and long, extending over tergite 9 apically, aedeagus small, star-shaped.

##### Description.

**Male.** Body length 2.7–2.8 mm (n = 2). ***Coloration*.** Head with vertex and frons dark brown, face and clypeus brown and mouthparts including palpus pale yellow. Scape and pedicel yellow. First three or four flagellomeres yellowish, rest of flagellomeres light brown. Scutum entirely dark brown, antepronotum and proepisternum yellowish, anepisternum, anepimeron and katepisternum light brown, laterotergite and mediotergite brown, scutellum brown. Thoracic setae all yellowish. Wing hyaline, all veins brown including radial veins somewhat darker. Halter with stem and knob pale yellow. All coxae, femora and tibiae yellow, tarsi yellow but seem darker because of dense brown setae. Tibial setae brown, spurs yellowish. Abdomen with tergites light brown, 1–3 tergites somewhat lighter, all sternites yellowish. Abdominal vestiture yellow. Terminalia brown. ***Head*.** Ocelli in a shallow triangular arrangement. Medial ocellus somewhat smaller than laterals. Frontal furrow complete. Clypeus subrounded, about as long as broad. Fourth flagellar segment about as long as wide, apical flagellar segment 2.25 times as long as wide basally. Flagellar segments with dense yellowish short setae. ***Thorax*.** Scutum covered with short setae, with marginal and prescutellar setae stronger. Antepronotum with 8–9 setae. Proepisternum with 6–7 setae. Anepisternum with 5–6 setae on upper part, katepisternum and anepimeron non-setose. Laterotergite with 7–9 setae on posterior half. Mediotergite with 10–15 setae on lower part. Metepisternum with setulae. Scutellum with setulae and marginal setae not arranged in pairs. ***Wing*.** Length 2.5–2.8 mm, length to width 2.4–2.7. Wing membrane uniformly covered with micro- and macrothichia. All veins setose, except *sc-r*, *Rs*, *R_2+3_*. Costa reaches about one fifth from *R_4+5_* to *M_1_*. *Sc* ending on *C* before level of furcation of posterior fork. *Sc-r* located slightly before *Rs. r-m* about two times as long as *m-stem*. *M_4_* basally very faint or shortly interrupted at base. ***Legs*.** Ratio of femur to tibia for fore, mid and hind legs: 0.83–0.93; 0.89–0.97; 0.84–0.92. Ratio of tibia to basitarsus for fore, mid and hind legs: 1.26; 1.42–1.65; 1.33–1.37. Fore tibia with a spur 2.29–2.81 times of tibial maximum width. Mid tibia with anterior spur 3.08–3.15 times and posterior spur 3.42–3.69 times of tibial maximum width. Hind tibia with anterior spur 2.50–2.60 times and posterior spur 3.47–3.57 times of tibial maximum width. ***Terminalia*** (Fig. 4A–G). Gonocoxites fused for short distance ventrobasally forming medial triangular lobe with medial more sclerotized longitudinal ridge internally. Ventromedial margin of gonocoxite with a membranous flange drawn medially out into digitate apically hooked lobe. Gonocoxite covered with uniform setae except non-setose lateroapical and dorsomedial marginal areas. Dorsoposterior margin of gonocoxite with two prominent medially directed setae. Gonocoxal apodeme anteriorly enlarged, shoe-shaped, well discernible in dorsal view. Tergite 9 large, slightly convergent posteriorly, extending over gonocoxites, subapically constricted with two prominent simple setae apically, apical margin medially rising. Parameres long and straight, apically slightly widening, extending over tergite 9 apically. Aedeagus small, star-shaped, medially with posteriorly projecting digitate process. Lateral branch of the gonostylus laterally setose with aggregation of spine like setae along posterior margin; ventral part extended with two prominent apical spine-like setae. Medial branch of gonostylus with 25–30 furcated megasetae. Small internal branch of gonostylus with one prominent medially directed seta.

**Figure 3.**
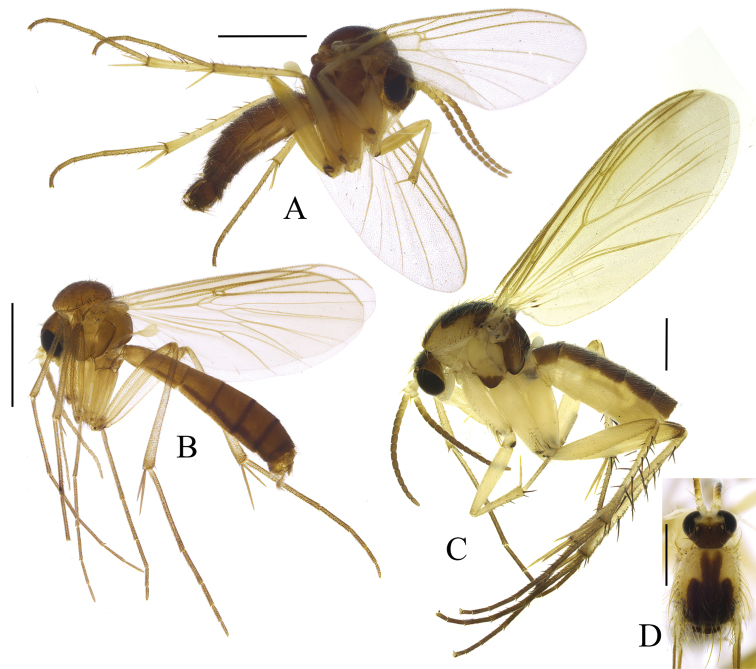
Habitus photos of new species **A***Sciophila georgei* sp. nov., paratype **B***Anatella metae* sp. nov., paratype **C***Leia katae* sp. nov., holotype, terminalia detached. Scale bars: 1 mm.

**Female.** Unknown.

##### Etymology.

The species is named in honour of Prof. George Japoshvili (Tbilisi, Georgia) in recognition of his contribution to study of the insects’ diversity in Georgia and his invaluable help in collecting the fungus gnat material that underlies the current communication. He was also the collector of the type material of this species.

**Figure 4.**
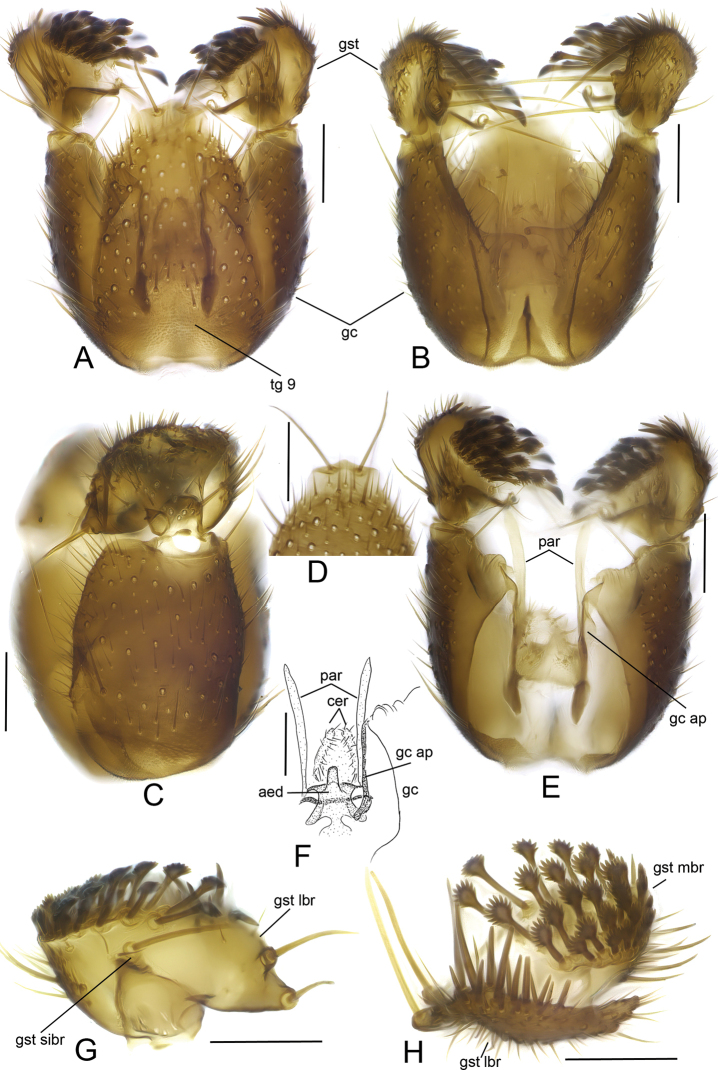
*Sciophila georgei* sp. nov., male terminalia **A** dorsal view **B** ventral view **C** lateral view **D** apical part of tergite IX, dorsal view **E** dorsal view, tergite IX removed **F** aedeagal complex, dorsal view **G** gonostylus, internal view **H** gonostylus, posterior view. Abbreviations: aed = aedeagus, cer = cercus, gc = gonocoxite, gc ap = gonocoxal apodeme, gst lbr = lateral branch of gonostylus, gst mbr = medial branch of gonostylus, gst sibr = small internal branch of gonostylus, par = paramere, tg 9 = tergite IX. Scale bars: 0.1 mm.

##### Remarks.

More than 190 species of *Sciophila* Meigen are known wordwide (Kurina 2020a, Taber 2021); the most comprehensive key to the Holarctic species is still that by Zaitzev (1982). Fortunately, all subsequently described species are supplemented with appropriate illustrations of the male terminalia (e.g. Polevoi 2001; Salmela and Kolcsár 2017; Taber 2021) that provides an adequate compendium of the morphological distinctions. Following the key by Zaitzev (1982), the new species runs to couplet 31 because of (1) wing with both macro- and microtrichia, (2) gonostylus without additional branches, (3) lateral branch of the gonostylus with two large setae ventroapically, (4) small internal branch of the gonostylus with one large seta, and (5) gonocoxites dorsoapically without protruding appendages. However, *S. georgei* sp. nov. differs from the species included in this couplet by details in the male terminalia. Notably, by the characters of tergite 9 (large, posteriorly convergent, extends over gonocoxites, bears two prominent simple setae apically, apical margin medially rising) and parameres (long, straight, extending over tergite 9 apically).

#### Leia katae sp. nov.

Taxon classificationAnimaliaDipteraMycetophilidae

69C08F18-98A3-5FC4-B902-31C0E6026560

http://zoobank.org/FDD299DF-4281-4DD0-9BAC-8B6050D98226

**Figure 5.**
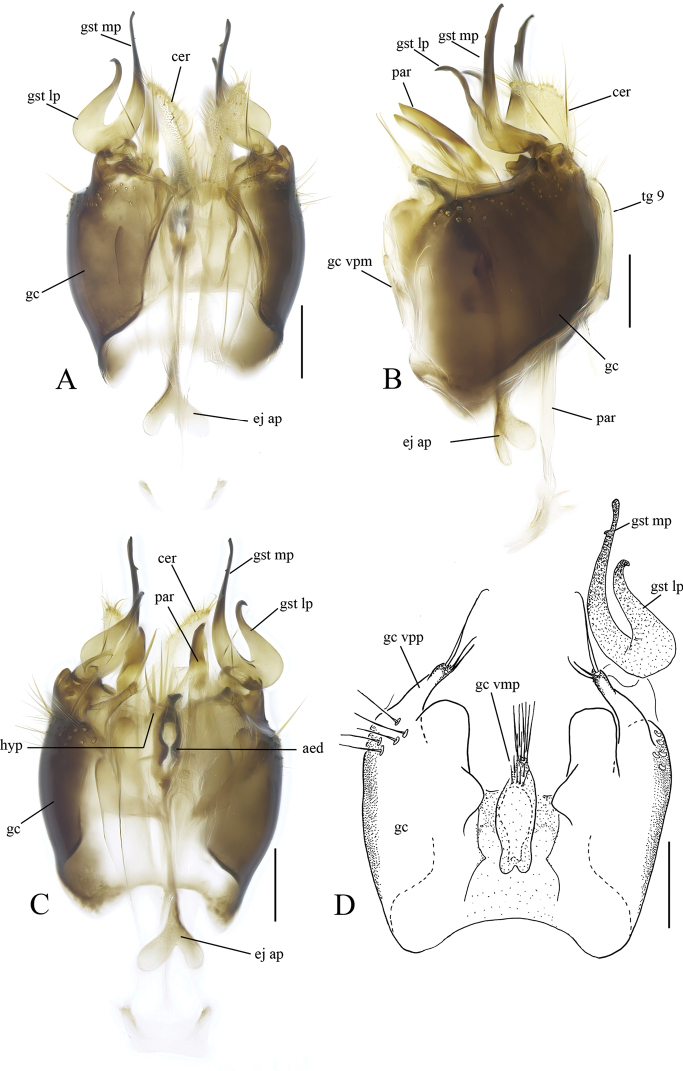
*Leia katae* sp. nov., male terminalia **A** dorsal view **B** lateral view **C** ventral view **D** gonocoxite and gonostylus, ventral view. Abbreviations: aed = aedeagus, cer = cercus, ejap = ejaculatory apodeme, gc = gonocoxite, gc vmp = ventromedial process of gonocoxite, gc vpp = ventroposterior process of gonocoxite, gst lp = lateral prong of gonostylus, gst mp = medial prong of gonostylus, hyp = hypoproct, par = paramere, tg 9 = tergite IX. Scale bars: 0.2 mm.

**Figure 6.**
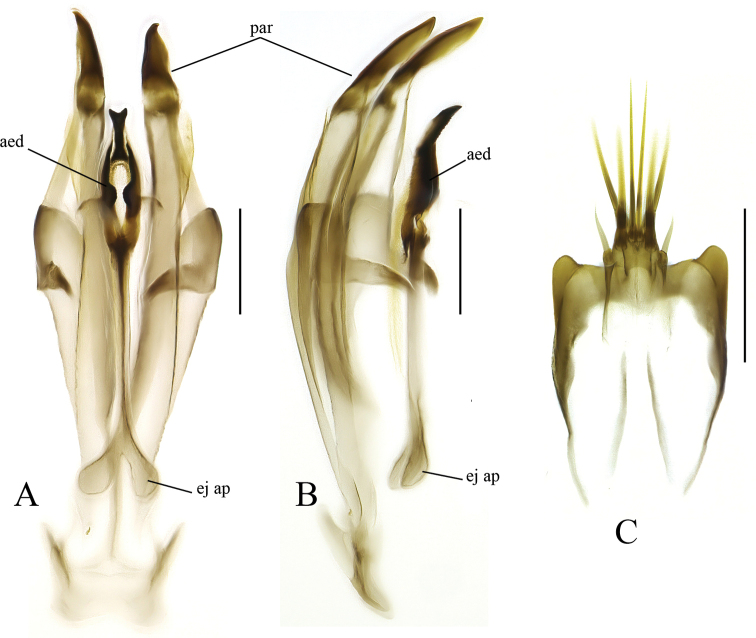
*Leia katae* sp. nov., male terminalia **A** aedeagal complex, ventral view **B** aedeagal complex, lateral view **C** hypoproct ventral view. Abbreviations: aed = aedeagus, ejap = ejaculatory apodeme, par = paramere. Scale bars: 0.2 mm.

Figs 3C, D , 5A–D , 6A–C

##### Type material.

***Holotype*.** Male, Georgia, Shida-Kartli, W of Surami, 42°01.57'N, 43°29.88'E, 940 m a.s.l., 18.v.2013, sweeping, leg. O. Kurina [see Table 1: SK-1] (mounted from alcohol, IZBE). ***Paratype*.** Male, GEORGIA, Samegrelo-Zemo-Svanethi, near Ushguli, path to glacier, 42°56.62'N, 43°03.23'E, 2220 m a.s.l., 15–17.vi.2019, Malaise trap, leg. X. Mengual [see Table 1: SZS-4] (in alcohol, ZFMK)

##### Diagnosis.

*Leia katae* sp. nov. can be distinguished by the combination of characters as follows: thorax bicolored (scutum yellow, with brown longitudinal stripes; katepisternum with lower half brown), wing tinged yellowish, with faint preapical brownish band, male terminalia with bipartite gonostylus (lateral prong shorter, convolute and apically hooked; medial prong longer, tapering with preapical small tooth at ventral margin).

##### Description.

**Male.** Body length 6.7–6.9 mm (n = 2). ***Coloration*.** Head with vertex brown, frons yellow, face, clypeus and mouthparts including palpus pale yellow. Scape and pedicel pale yellow. First two flagellomeres yellowish, flagellomeres 3–14 brown. Thorax bicoloured: scutum yellow with three brown longitudinal stripes, which are posteriorly completely fused, lateral stripes begin at a distance of one third from anterior margin, medial stripe shortly split anteriorly, lateral parts of scutum yellow; antepronotum, proepisternum and anepisternum yellow, posterior margin of anepimeron light brown, katepisternum with lower half brown and upper half yellow, laterotergite brown with posterior half yellowish, mediotergite brown, scutellum basally yellowish, apically brown. Thoracic setae all yellow. Wing with yellowish tinge and preapical very faint transverse brownish band reaching to *M_2_*, all veins yellowish including radial veins somewhat darker. Halter with stem and knob pale yellow. All coxae and femora yellow, except hind femur apically with narrow brown band, all tibiae, tarsi yellow but seem darker because of dense brown setae. Tibial setae brown, spurs yellowish. Abdomen with all tergites brown and sternites yellow. Abdominal vestiture yellow. Terminalia brown with gonocoxite medially and gonostylus anteriorly yellow. ***Head*.** Ocelli in a linear arrangement. Medial ocellus about twice smaller than laterals, which are separated from eye margins by less than their own diameter. Frontal furrow complete. Clypeus obovoid. Fourth flagellar segment about as long as wide, apical flagellar segment 2.5 times as long as wide basally. Flagellar segments with dense yellowish short setae. ***Thorax*.** Scutum densely covered with setae, with marginal and prescutellar setae stronger. Antepronotum with 6–7 strong and a number of weaker setae. Proepisternum with one very strong seta at anterior margin about 10 weaker setae. Anepisternum, katepisternum and anepimeron non-setose. Laterotergite with long fine setae on posterior half. Mediotergite non-setose. Scutellum with a row of marginal setae including two pairs remarkably stronger. ***Wing*.** Length 5.3–5.7 mm, length to width 2.3–2.8. All veins setose, except *Sc*, *sc-r*, *Rs* and extreme base of *M_1_*. *Sc* ending on *C* at level of furcation of posterior fork. *R_4+5_* 3.3 times as long as *R*_1_. *r-m* 1.47 times as long as *m-stem*. *M_1_* and *M_2_* apically convergent, apical third of both veins faint. *M_4_* interrupted at base. *Rs* located distally of the anterior fork. ***Legs*.** Ratio of femur to tibia for fore, mid and hind legs: 1.16; 0.95; 0.86. Ratio of tibia to basitarsus for fore, mid and hind legs: 1.00; 1.66; 2.00. Fore tibia with a spur 2.95 times of tibial maximum width. Mid tibia with anterior spur 3.33 times and posterior spur 3.96 times of tibial maximum width. Hind tibia with anterior spur 3.33 times and posterior spur 4.58 times of tibial maximum width. ***Terminalia*** (Figs 5A–D, 6A–C). Gonocoxite with setae on apical fifth only. Ventromedial process of gonocoxite elongated ovoid with a row of long setae apically and an aggregation of shorter setae on apical fourth. Ventroposterior margin of gonocoxite drawn into a wide membraneous non-setose medial lobe and a digitate more protruding lobe with one prominent and 2–3 weaker apical setae. Tergite 9 membraneous, somewhat tapering, apically evenly rounded with apicocentral patch of short setae. Gonostylus bipartite: lateral prong shorter, convolute and apically hooked; medial prong longer, tapering with preapical small tooth at ventral margin. Aedeagus with sclerotized, cup-shaped apical portion, ejaculatory apodeme bilobed. Paramere about 1.6 times as long as aedeagus, bowed in lateral view, apically tapering, with ventral flange drawn out into a triangular membranous process in the middle; anteriorly, parameres fused into a complex membranous structure with anterior concavity and well protruding lateral corners. Hypoproct with protruding apicolateral corners and medial part that bears a group of stout setae.

**Female.** Unknown.

##### Etymology.

The species is named after my daughter Katariina (born 3 November 2000), an enthusiastic student of biology at the University of Tartu (Estonia). She participated in a trip to Georgia in 2017 that yielded several specimens of this study and she always insists we call her Kata.

##### Remarks.

There are 166 *Leia* Meigen species known worldwide including 33 in the Palaearctic region (Polevoi and Salmela 2016). *Leia katae* sp. nov. differs from all known Palaearctic and Nearctic species by its peculiar structure of the gonostylus that is bipartite: medial prong long and slender with a preapical tooth, and lateral prong apically hooked, about 2/3 of the medial prong.

#### Anatella metae sp. nov.

Taxon classificationAnimaliaDipteraMycetophilidae

C5110FF4-1D4E-5E05-9253-4370412A76A0

http://zoobank.org/7CD91319-0672-4DC8-9CF6-7140ADF1F13E

Figs 3B , 7A–G

##### Type material.

***Holotype*.** Male, Georgia, Mtskhetha-Mthianethi, Saguramo north of Tbilisi, 41°53.07'N, 44°46.78'E, 920 m a.s.l., 15.v.2013, sweeping, leg. O. Kurina [see Table 1: MM-12] (mounted from alcohol, IZBE). ***Paratype*.** Male, same as holotype (mounted from alcohol, IZBE).

##### Diagnosis.

*Anatella metae* sp. nov. is characterized by the presence of a strong posteroventral fringe on mid femora with a row of strong setae, absence of anterior spur on mid tibia, absence of setae on hind coxa basally. The new species is closest to *A. atlanticiliata* Chandler and Ribeiro but differs in characters of the male terminalia: ventral branch of the gonostylus about twice as long as the dorsal branch, dorsal branch of the gonostylus with long and slender medial prong, medial branch of the gonostylus slender and apically hooked.

##### Description.

**Figure 7.**
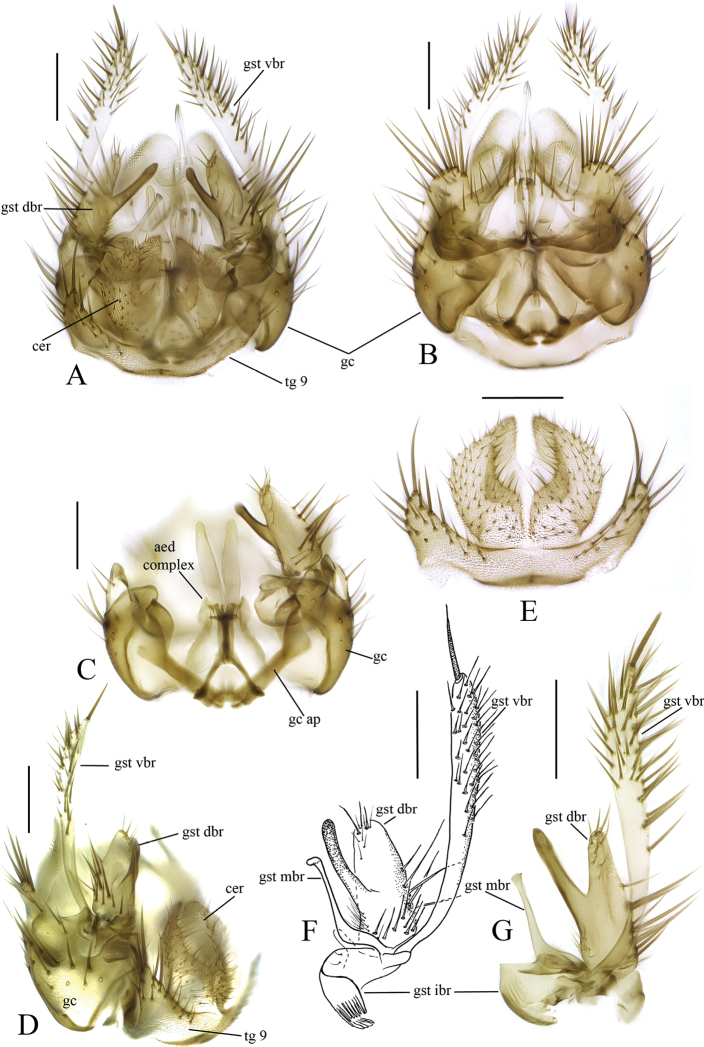
*Anatella metae* sp. nov. male terminalia **A** dorsal view **B** ventral view **C** dorsal view, cerci and tergite IX removed **D** lateral view **E** cerci and tergite IX, dorsal view **F, G** gonostylus, internal views from different angles. Abbreviations: aed complex – aedeagal complex, cer = cercus, gc = gonocoxite, gc ap = gonocoxal apodeme, gst dbr = dorsal branch of gonostylus, gst ibr = internal branch of gonostylus, gst mbr = medial branch of gonostylus, gst vbr = ventral branch of gonostylus, tg 9 = tergite IX. Scale bars: 0.1 mm.

**Male.** Body length 2.7–2.9 mm (n = 2). ***Coloration*.** Head with vertex, frons, face and clypeus brown, mouthparts including palpus pale yellow. Scape, pedicel and base of first flagellomere yellow, rest of flagellum light brown. Thorax with scutum and lateral parts light brown. Thoracic setae yellowish to brown, with thicker setae darker than finer ones. Wing hyaline, unmarked with yellowish tinge. Halter with stem and knob pale yellow. Legs yellow, tarsi yellow but seem darker because of dense brown setae. All setae on legs brown, tibial spurs yellowish. Abdomen mainly brown with first two segments somewhat lighter. Abdominal vestiture brown. Terminalia light brown. ***Head*.** Ocelli two, very close to eye margins, with dark brown patches at anterior margin. Frontal furrow complete. Clypeus rectangular. Fourth flagellar segment about 2.5 times as long as wide, apical flagellar segment 2.5 times as long as wide basally. Flagellar segments with dense whitish setae about one third of segments’ width. ***Thorax*.** Scutum covered with setae, with marginal and prescutellar setae stronger. Antepronotum with 2 strong and 10–15 weaker setae. Proepisternum with two strong and 2–3 weaker setae. Anepisternum, katepisternum and anepimeron non-setose. Laterotergite with about 10 setae on upper half. Mediotergite non-setose. Scutellum with about 10 setae on upper surface. ***Wing*.** Length 2.39–2.70 mm, length to width 2.75–2.90. *C*, *R*, *R_1_*, *R_4+5_* setose, all other veins non-setose. *C* produced halfway between *R_4+5_* and *M_1_*. *r-m* about as long as *m-stem*. Posterior fork at the level of anterior fork or slightly before. *CuA* slightly sinuous. ***Legs*.** Ratio of femur to tibia for fore, mid and hind legs: 1.08–1.17; 0.97–1.00; 0.65–0.90. Ratio of tibia to basitarsus for fore, mid and hind legs: 0.96–1.00; 1.21–1.28; 1.41–1.77. Fore tibia with a spur 2.00 times of tibial maximum width. Mid tibia with anterior spur absent and posterior spur 2.27–2.40 times of tibial maximum width. Hind tibia with anterior spur 2.71–3.33 times and posterior spur 3.93–4.66 times of tibial maximum width. Strong posteroventral fringe of mid femora with row of strong setae. Hind coxa without basal setae. ***Terminalia*** (Fig. 7A–G). Gonocoxite ventrally with (1) V-shaped wide incision anteriorly, (2) posteromedial non-setose tapering projection with deep slit, and (3) posterolateral large apically setose lobes. Gonostylus divided into four branches (Fig. 7F–G). The ventral branch of the gonostylus elongated digitate, apical half setose and with one strong seta apically deviating from other setosity. Dorsal branch of the gonostylus about half length of the ventral branch, divided into two prongs: medial finger like bare prong and lateral large apically and basally setose prong. Medial branch of the gonostylus slightly shorter than dorsal branch, slender, apically hooked. Internal branch of the gonostylus membranous, convolute with anterior lamellae.

##### Etymology.

The species is named after my daughter Liisa-Meta (born 9 October 2004), a keen naturalist who also participated in a trip to Georgia in 2017.

##### Remarks.

There are about 50 *Anatella* Winnertz species known in the Holarctic region (cf. Fungus Gnats Online Authors 2021), the vast majority of which are adequately described and figured. In having posteroventral fringe of mid femora with strong setae and absence of anterior spur of mid tibia, *A. metae* sp. nov. resembles *A. atlanticiliata* Chandler & Ribeiro, 1995 known only from Madeira Island. Also, the male terminalia of both species share the general outline (cf. Chandler and Ribeiro 1995: fig. 27). However, *A. metae* differs in the structure of the gonostylus as follows: (1) ventral branch of the gonostylus twice as long as dorsal branch (only somewhat longer in *A. atlanticiliata*), (2) dorsal branch of the gonostylus with medial prong long, anchored to lateral prong basally (medial prong short, anchored to lateral prong subapically in *A. atlanticiliata*), and (3) medial branch of gonostylus slender, apically hooked (medial branch stout, slightly curved in *A. atlanticiliata*).

### List of fungus gnat species of Georgia

#### Family Bolitophilidae

##### 1. Bolitophila (Bolitophila) austriaca

Taxon classificationAnimaliaDipteraBolitophilidae

(Mayer, 1950)

760DD7E7-5BF5-52A2-9515-0D090CDCB1BD

###### Material.

2♀♀, SJ-7; 4♀♀, SJ-8. Total: 6♀♀.

###### Distribution in Georgia.

Samtskhe-Javakheti.

###### General distribution.

Palaearctic.

##### 2. Bolitophila (Bolitophila) basicornis

Taxon classificationAnimaliaDipteraBolitophilidae

(Mayer, 1951)

585C6050-A66B-5B25-92E7-11A9C3C68BB6

Fig. 9A

###### Material.

1♀, MM-1. Total: 1♀.

###### Distribution in Georgia.

Mtskhetha-Mthianethi.

###### General distribution.

Palaearctic.

##### 3. Bolitophila (Bolitophila) cinerea

Taxon classificationAnimaliaDipteraBolitophilidae

Meigen, 1818

36A13E06-BFD7-577F-80EB-E6664FDB2E5A

###### Material.

1♂, MM-12. Total: 1♂.

###### Distribution in Georgia.

Mtskhetha-Mthianethi.

###### General distribution.

Palaearctic.

##### 4. Bolitophila (Cliopisa) fumida

Taxon classificationAnimaliaDipteraBolitophilidae

Edwards, 1941

E764C905-F852-599E-8352-2F912BC50FD7

###### Material.

1♂, SJ-9. Total: 1♂.

###### Distribution in Georgia.

Samtskhe-Javakheti.

###### General distribution.

Palaearctic.

#### Family Diadocidiidae

##### 5. Diadocidia (Adidocidia) valida

Taxon classificationAnimaliaDipteraDiadocidiidae

Mik, 1874

82387C38-2B44-5221-8C79-939284E382E4

###### Material.

1♀, SJ-1; 1♂, SK-1. Total: 1♂ 1♀.

###### Distribution in Georgia.

Shida Kartli, Samtskhe-Javakheti.

###### General distribution.

Western Palaearctic.

###### Remarks.

In Transcaucasia recorded from Azerbaijan (Zaitzev 1994).

##### 6. Diadocidia (Diadocidia) ferruginosa

Taxon classificationAnimaliaDipteraDiadocidiidae

(Meigen, 1830)

8C209075-9BBA-5FDF-90AC-22FEBB07089E

###### Material.

1♂, SZS-3 (ZFMK); 2♂♂ 1♀, I-6; 2♂♂, I-9; 1♂, I-11; 1♂, A-1; 1♂, SJ-4; 1♂, SJ-7; 1♂, SJ-8; 4♂♂, SK-1. Total: 14♂♂ 1♀.

###### Distribution in Georgia.

Samegrelo-Zemo Svanethi, Shida Kartli, Imereti, Samtskhe-Javakheti.

###### General distribution.

Holarctic.

##### 7. Diadocidia (Diadocidia) spinosula

Taxon classificationAnimaliaDipteraDiadocidiidae

Tollet, 1948

8562BA00-C871-56E8-9A6F-4B53E0C6244C

Fig. 8G

###### Material.

1♂, SZS-3 (IZBE); 2♂♂, SK-1; 2♂♂, SJ-1 (ZFMK); 1♂, SJ-2 (IUTG); 1♂ 1♀, SJ-4; 2♂♂, SJ-7; 4♂♂ 3♀♀, SJ-9. Total: 13♂♂ 4♀♀.

###### Distribution in Georgia.

Samegrelo-Zemo Svanethi, Shida Kartli, Samtskhe-Javakheti.

###### General distribution.

Palaearctic.

**Figure 8.**
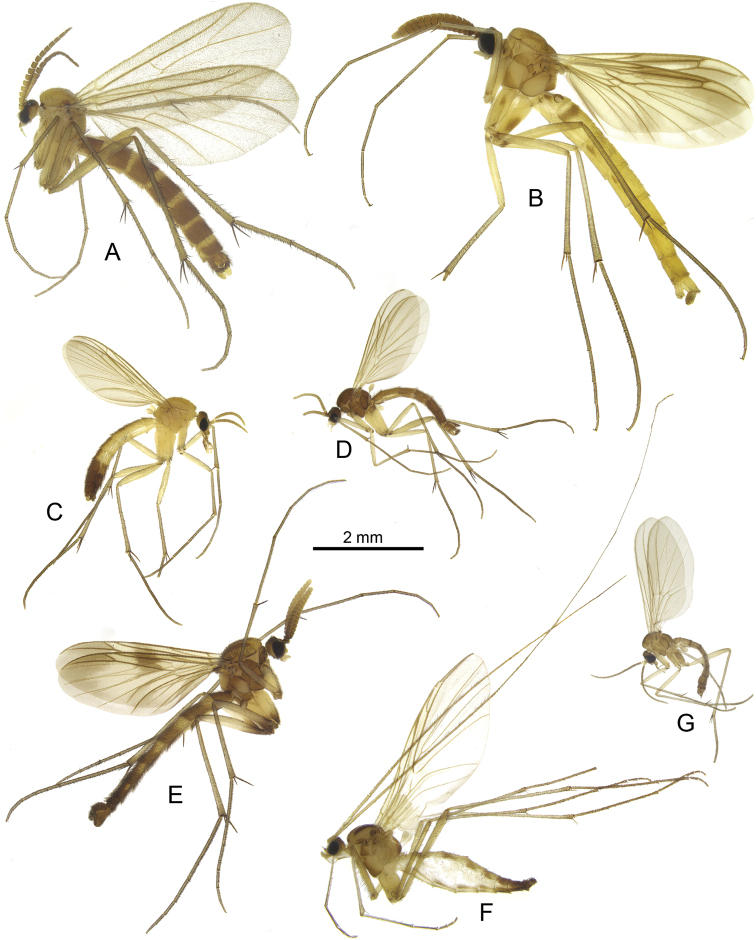
Habitus of Georgian fungus gnats of the families Ditomyiidae (**A**), Keroplatidae (**B–F**) and Diadocidiidae (**G**) **A***Symmerus annulatus* (Meigen, 1830) **B***Keroplatus testaceus* Dalman, 1818 **C***Macrorrhyncha flava* Winnertz, 1846 **D***Pyratula zonata* (Zetterstedt, 1855) **E***Cerotelion racovitzai* Matile & Burghele-Balacesco, 1969 **F***Macrocera vittata* Meigen, 1830 **G**Diadocidia (Diadocidia) spinosula Tollet, 1948.

#### Family Ditomyiidae

##### 8. Ditomyia fasciata

Taxon classificationAnimaliaDipteraDitomyiidae

(Meigen, 1818)

2DB9C662-7DE3-589A-8C0D-E962AAC24EAC

###### Material.

2♂♂, I-9. Total: 2♂♂.

###### Distribution in Georgia.

Imereti.

###### General distribution.

Palaearctic.

###### Remarks.

In Transcaucasia recorded from Azerbaijan (Zaitzev 1994).

##### 9. Symmerus annulatus

Taxon classificationAnimaliaDipteraDitomyiidae

(Meigen, 1830)

BC071DFE-4E2F-5EED-AE26-506DC6BEBC29

Fig. 8A

###### Material.

6♂♂, SZS-3 (2♂♂ ZFMK, 2♂♂ IUTG, 2♂♂ IZBE); 1♂, A-1; 1♂ 1♀, A-3; 1♀, I-3 (18.v–1.vi.2013); 1♂, I-6; 1♂ 1♀, I-9; 1♂, I-10; 2♂♂, I-11; 1♂, I-12; 4♂♂, SJ-1 (2♂♂ ZFMK, 1♂ IUTG, 1♂ IZBE); 2♂♂, MM-12. Total: 20♂♂ 3♀♀.

###### Distribution in Georgia.

Samegrelo-Zemo Svanethi, Adjara, Imereti, Samtskhe-Javakheti, Mtskhetha-Mthianethi.

###### General distribution.

Palaearctic.

###### Remarks.

In Transcaucasia recorded from Azerbaijan (Zaitzev 1994).

#### Family Keroplatidae

**Subfamily Macrocerinae**

##### 10. Macrocera centralis

Taxon classificationAnimaliaDipteraKeroplatidae

Meigen, 1818

CD6790A9-515B-57A3-BA08-9C3444663E2E

###### Material.

2♂♂, KK-1; 1♀, MM-14. Total: 2♂♂ 1♀.

###### Distribution in Georgia.

Kvemo Kartli, Mtskhetha-Mthianethi.

###### General distribution.

Palaearctic.

##### 11. Macrocera crassicornis

Taxon classificationAnimaliaDipteraKeroplatidae

Winnertz, 1863

CCAA9AB2-C153-507B-9A41-E04DF1CE60A9

###### Material.

1♂, A-1. Total: 1♂.

###### Distribution in Georgia.

Adjara.

###### General distribution.

Palaearctic.

###### Remarks.

In Transcaucasia recorded from Armenia (Zaitzev 1994).

##### 12. Macrocera fasciata

Taxon classificationAnimaliaDipteraKeroplatidae

Meigen, 1804

4B472653-2A08-537E-B6E7-AA5D8493EC19

###### Material.

1♂, SZS-3 (ZFMK). Total: 1♂.

###### Distribution in Georgia.

Samegrelo-Zemo Svanethi.

###### General distribution.

Palaearctic.

##### 13. Macrocera fastuosa

Taxon classificationAnimaliaDipteraKeroplatidae

Loew, 1869

43A949F8-3940-5B53-9682-38EE8B11B229

###### Material.

2♂♂, A-1; 3♂♂, A-7. Total: 5♂♂.

###### Distribution in Georgia.

Adjara.

###### General distribution.

Europe.

##### 14. Macrocera lutea

Taxon classificationAnimaliaDipteraKeroplatidae

Meigen, 1804

B05D00CF-84B4-5A68-B7EB-261EA8661ECF

###### Material.

1 ♂, A-7; 1♂, KK-1. Total: 2♂♂.

###### Distribution in Georgia.

Adjara, Kvemo Kartli.

###### General distribution.

Palaearctic.

###### Remarks.

In Transcaucasia recorded from Armenia (Joost and Plassmann 1985).

##### 15. Macrocera phalerata

Taxon classificationAnimaliaDipteraKeroplatidae

Meigen, 1818

92A6D5F2-350E-570E-815A-37710616EBF3

###### Material.

1♀, A-1; 1♀, A-6; 1♀, A-7. Total: 3♀♀.

###### Distribution in Georgia.

Adjara.

###### General distribution.

Palaearctic.

##### 16. Macrocera stigma

Taxon classificationAnimaliaDipteraKeroplatidae

Curtis, 1837

CB1460A1-ABDF-568D-AADE-FDBCC2F282B1

###### Material.

1♂ 1♀, A-1; 4♂♂, A-7; 11♂♂ 3 ♀♀, I-6; 2♂♂, I-11; Total: 18♂♂ 8♀♀.

###### Distribution in Georgia.

Adjara, Imereti.

###### General distribution.

Palaearctic.

##### 17. Macrocera stigmoides

Taxon classificationAnimaliaDipteraKeroplatidae

Edwards, 1925

12DAB285-B13B-51F7-8D6D-F2D1CD26F28B

###### Material.

44♂♂ 14♀♀, KK-1. Total: 44♂♂ 14♀♀.

###### Distribution in Georgia.

Kvemo Kartli.

###### General distribution.

Palaearctic.

##### 18. Macrocera vittata

Taxon classificationAnimaliaDipteraKeroplatidae

Meigen, 1830

1D1CAFBE-951D-5103-BE4A-2F5634C9317F

Fig. 8F

###### Material.

3♂♂, SJ-8; 2♂♂, MM-13; 1♂, MM-14. Total: 6♂♂.

###### Distribution in Georgia.

Samtskhe-Javakheti, Mtskhetha-Mthianethi.

###### General distribution.

Palaearctic.

#### Subfamily Keroplatinae

**Tribe Keroplatini**

##### 19. Cerotelion racovitzai

Taxon classificationAnimaliaDipteraKeroplatidae

Matile & Burghele-Balacesco, 1969

BEBA1448-A91A-5BF9-8292-C139F59B64CE

Fig. 8E

###### Material.

6♂♂, A-3; 1♂, A-5; 3♂♂, A-7; 14♂♂ 1♀, I-6; 1♀, I-8; 3♂♂, I-9; 1♂, I-10; 2♂♂, I-11; 2♂♂, I-14; 1♂, I-15; 1♀, MM-7; 3♂♂, MM-8. Total: 36♂♂ 3♀♀.

###### Distribution in Georgia.

Adjara, Imereti, Mtskhetha-Mthianethi.

###### General distribution.

Western Palaearctic.

###### Remarks.

In Transcaucasia recorded from Azerbaijan (Zaitzev 1994).

##### 20. Cerotelion striatum

Taxon classificationAnimaliaDipteraKeroplatidae

(Gmelin, 1790)

5296B60A-9F13-5F7A-982F-2A72AFA5AC30

###### Material.

1♂, I-4. Total: 1♂.

###### Distribution in Georgia.

Imereti.

###### General distribution.

Western Palaearctic.

###### Remarks.

In Transcaucasia recorded from Azerbaijan (Zaitzev 1994).

##### 21. Keroplatus testaceus

Taxon classificationAnimaliaDipteraKeroplatidae

Dalman, 1818

EF3C443B-C58B-5F26-BDBD-63FEC08B7522

Fig. 8B

###### Material.

2♂♂, I-6. Total: 2♂♂.

###### Distribution in Georgia.

Imereti.

###### General distribution.

Palaearctic.

###### Remarks.

In Transcaucasia recorded from Azerbaijan (Zaitzev 1994).

#### Tribe Orfeliini

##### 22. Isoneuromyia semirufa

Taxon classificationAnimaliaDipteraKeroplatidae

(Meigen, 1818)

95212B05-5ABD-5448-9D72-3FF4B76F9C05

###### Georgian source.

Zaitzev 1994: 82 (from Adjara).

###### Distribution in Georgia.

Adjara.

###### General distribution.

Holarctic.

###### Remarks.

Zaitzev (1994) studied a single male specimen from Batumi collected in 1908. The black colour of the body as noted by Zaitzev (1994) for the studied material is characteristic to *I. semirufa*. The other European species have the thorax yellow to orange with or without longitudinal stripes; also, see the next species and discussion by Mantič and Ševčík (2017).

##### 23. Isoneuromyia czernyi

Taxon classificationAnimaliaDipteraKeroplatidae

(Strobl, 1909)

C50699F7-6CAE-5CAA-BCBE-D065A50339DD

###### Material.

1♂, A-1; 1♂, A-5; 1♀, A-7; 1♂ 1♀, I-6. Total: 3♂♂ 2♀♀.

###### Distribution in Georgia.

Adjara, Imereti.

###### General distribution.

Europe.

###### Remarks.

All studied Georgian specimens correspond to the diagnosis including figures provided recently by Mantič and Ševčík (2017), i.e. (1) scutum with longitudinal dark stripes which are, however, almost fused in female specimens (thorax all dark brown to blackish in *I. semirufa*), (2) wing with a distinct subapical band (anteriorly infuscated in *I. semirufa*) and (3) male terminalia with medial tooth of the gonostylys larger than the lateral one (both in subequal size in *I. semirufa*). *I. czernyi* is a rare European species known from the Mediterranean region and Slovakia (Mantič and Ševčík 2017).

##### 24. Macrorrhyncha flava

Taxon classificationAnimaliaDipteraKeroplatidae

Winnertz, 1846

0FCF7936-19E5-565F-BF86-D2A91CC2190F

Fig. 8C

###### Material.

2♂♂, K-4; 7♂♂ 3♀♀, K-5. Total: 9♂♂ 3♀♀.

###### Distribution in Georgia.

Kakheti.

###### General distribution.

Europe.

##### 25. Monocentrota lundstromi

Taxon classificationAnimaliaDipteraKeroplatidae

Edwards, 1925

7F2F4C3E-8C86-5CF0-984D-4B14B4B8EBAB

###### Material.

1♂, SZS-3 (IZBE). Total: 1♂.

###### Distribution in Georgia.

Samegrelo-Zemo Svanethi.

###### General distribution.

Europe.

##### 26. Neoplatyura modesta

Taxon classificationAnimaliaDipteraKeroplatidae

(Winnertz, 1863)

D977DD28-F5E3-5DAE-A828-7572B2648E6E

###### Material.

1♂, K-2; 1♀, K-3. Total: 1♂ 1♀.

###### Distribution in Georgia.

Kakheti.

###### General distribution.

Europe.

##### 27. Neoplatyura nigricauda

Taxon classificationAnimaliaDipteraKeroplatidae

(Strobl, 1893)

5F94DBF1-DB7F-56F6-BE0D-11F82486C683

###### Material.

1♂, SZS-1. Total: 1♂.

###### Distribution in Georgia.

Samegrelo-Zemo Svanethi.

###### General distribution.

Europe.

##### 28. Orfelia discoloria

Taxon classificationAnimaliaDipteraKeroplatidae

(Meigen, 1818)

998EEC29-5151-5D99-B54D-1D832FCA78E5

###### Material.

7♂♂, SZS-3(2♂♂ ZFMK, 3♂♂ IUTG, 2♂♂ IZBE); 1♂, A-3; 1♂, SJ-1 (ZFMK); 1♂, MM-7. Total: 10♂♂.

###### Distribution in Georgia.

Samegrelo-Zemo Svanethi, Adjara, Samtskhe-Javakheti, Mtskhetha-Mthianethi.

###### General distribution.

Holarctic.

##### 29. Orfelia georgica

Taxon classificationAnimaliaDipteraKeroplatidae

Kurina & Jürgenstein, 2013

F0C5C228-45A1-54EC-95AA-BFC919D90CA6

Fig. 9B

###### Georgian source.

Kurina and Jürgenstein 2013: 23 (fig. 2a–d)

###### Type material.

1♂, I-10 (holotype); 1♂, I-9 (paratype); 10♂♂, I-6 (paratypes). Additional material. 12♂♂ 4♀♀, A-1; 34♂♂, A-3; 7♂♂, A-5; 76♂♂, A-7; 1♂, A-8; 5♂♂, I-6; 12♂♂, I-11; 1♂, I-12; 5♂♂, SJ-1; 1♂, SJ-2 (ZFMK); 4♂♂, K-4; 1♂, K-6. Total: 171♂♂ 4♀♀.

###### Distribution in Georgia.

Adjara, Imereti, Samtskhe-Javakheti, Kakheti.

###### General distribution.

Georgia.

**Figure 9.**
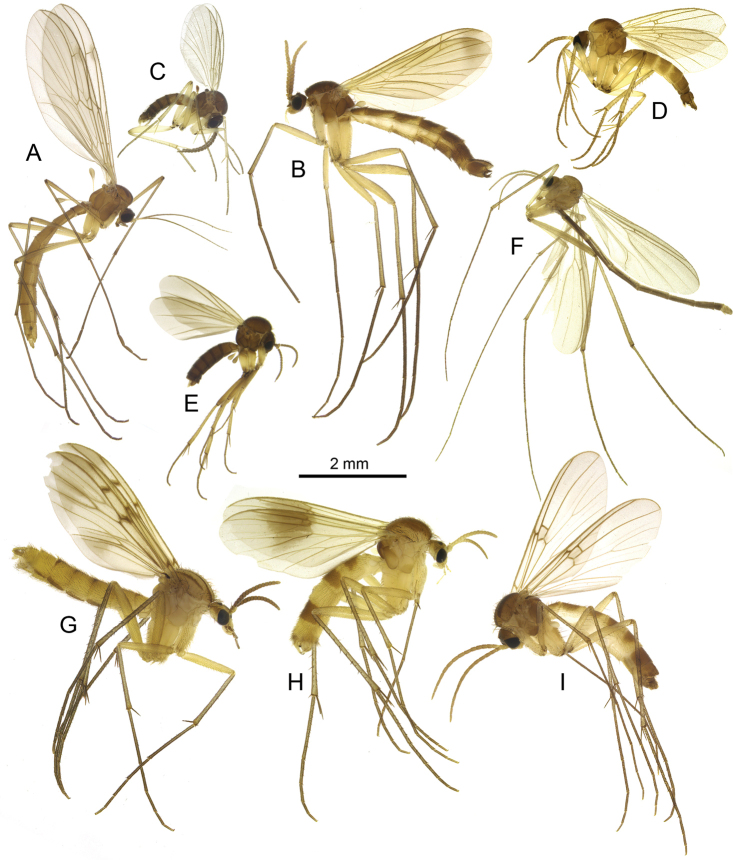
Habitus of Georgian fungus gnats of the families Bolitophilidae (**A**), Keroplatidae (**B**) and Mycetophilidae (**C–I**) **A**Bolitophila (Bolitophila) basicornis (Mayer, 1951) **B***Orfelia georgica* Kurina & Jürgenstein, 2013 **C***Acnemia nitidicollis* (Meigen, 1818) **D***Monoclona rufilatera* (Walker, 1837) **E**Azana (Azana) anomala (Staeger, 1840) **F***Phthinia hyrcanica* Zaitzev, 1984 **G***Neoempheria striata* (Meigen, 1818) **H***Neoempheria brevilineata* Okada, 1939 **I**Mycomya (Mycomya) marginata (Meigen, 1818).

##### 30. Orfelia trifida

Taxon classificationAnimaliaDipteraKeroplatidae

Kurina & Jürgenstein, 2013

0BF1E049-E613-58AF-BBDF-65B9FF21FB4B

###### Georgian source.

Kurina and Jürgenstein 2013: 24 (fig. 3a–d).

###### Type material.

1♂, I-5 (holotype). Additional material. 35♂♂, SZS-3 (12♂♂ ZFMK, 12♂♂ IUTG, 11♂♂ IZBE). Total: 36♂♂.

###### Distribution in Georgia.

Samegrelo-Zemo Svanethi, Imereti.

###### General distribution.

Georgia.

##### 31. Pyratula perpusilla

Taxon classificationAnimaliaDipteraKeroplatidae

(Edwards, 1913)

D65F51C5-DEAD-5EBD-87DA-F2440FE5794D

###### Material.

16♂♂, SJ-4. Total: 16♂♂.

###### Distribution in Georgia.

Samtskhe-Javakheti.

###### General distribution.

Europe.

###### Remarks.

The *P. perpusilla* species-group includes at least seven closely related species in Europe, separable only by small details of male terminalia (Chandler and Blasco-Zumeta 2001). The studied Georgian specimens have the ventroapical margin of the gonocoxite with setose lobe (= without asetose protuberance) that is shared by three species, viz. *P. perpusilla*, *P. alpicola* Chandler, 2001 and *P. oracula* Chandler, 1994. The aedeagal complex is considerably short (elongate in *P. oracula*) and the aedeagal sheath is interrupted medially on the ventral side (with complete bridge in *P. alpicola*). However, the Georgian specimens are slightly different from *P. perpusilla* as figured by Chandler and Blasco-Zumeta (2001: Figs 9–12) in having the distal dorsal corner of the aedeagal seath with a blunt protuberance that is otherwise typical to *P. alpicola*. The material was compared to that of *P. alpicola* and *P. oracula* from North Italy (cf. Kurina 2008b) and, pending a further molecular study of this species-group, is considered to be conspecific with *P. perpusilla*.

##### 32. Pyratula zonata

Taxon classificationAnimaliaDipteraKeroplatidae

(Zetterstedt, 1855)

0A8E2391-907C-5DF5-90C8-3CE5112A615C

Fig. 8D

###### Material.

2♂♂, A-5; 1♂, I-6; 2♂♂, I-11; 2♂♂, SJ-1 (1♂ IUTG, 1♂ IZBE); 1♂ 1♀, SJ-2 (ZFMK); 1♂, SJ-3; 4♂♂, MM-11. Total: 13♂♂ 1♀.

###### Distribution in Georgia.

Adjara, Imereti, Samtskhe-Javakheti, Mtskhetha-Mthianethi.

###### General distribution.

Europe.

##### 33. Urytalpa dorsalis

Taxon classificationAnimaliaDipteraKeroplatidae

(Staeger, 1840)

26C2DB5E-AFB6-5062-8F03-BF8B8116EF0F

###### Material.

1♂, SZS-3 (IZBE); 3♂♂, SJ-1 (1♂ ZFMK, 1♂ IUTG, 1♂ IZBE); 1♂ 2♀♀, SJ-2 (1♂ 1♀ ZFMK, 1♀ IUTG). Total: 5♂♂ 2♀♀.

###### Distribution in Georgia.

Samegrelo-Zemo Svanethi, Samtskhe-Javakheti.

###### General distribution.

Europe.

#### Subfamily Platyurinae

##### 34. Platyura marginata

Taxon classificationAnimaliaDipteraKeroplatidae

Meigen 1804

E331029B-412D-502E-A3B0-7F8DE1BA8B2E

###### Material.

1♂, K-6. Total: 1♂.

###### Distribution in Georgia.

Kakheti.

###### General distribution.

Palaearctic.

#### Family Mycetophilidae

**Subfamily Mycomyinae**

##### 35. Mycomya (Cymomya) circumdata

Taxon classificationAnimaliaDipteraMycetophilidae

(Staeger, 1840)

45B70A57-CAB3-5F1F-B32B-9ED35B4B091A

###### Material.

2♂♂, SZS-3 (1♂ ZFMK, 1♂ IZBE). Total: 2♂♂.

###### Distribution in Georgia.

Samegrelo-Zemo Svanethi.

###### General distribution.

Palaearctic.

##### 36. Mycomya (Neomycomya) fimbriata

Taxon classificationAnimaliaDipteraMycetophilidae

(Meigen, 1818)

1D8C5EF7-B16B-5F1F-A7BE-04702F696BAE

###### Material.

1♂, A-7. Total: 1♂.

###### Distribution in Georgia.

Adjara.

###### General distribution.

Holarctic, extending to the Oriental region.

##### 37. Mycomya (Mycomya) bialorussica

Taxon classificationAnimaliaDipteraMycetophilidae

Landrock, 1925

14E7A313-3C96-5BEB-9520-83526042BD61

###### Material.

1♂, SZS-3 (ZFMK). Total: 1♂.

###### Distribution in Georgia.

Samegrelo-Zemo Svanethi.

###### General distribution.

Europe.

##### 38. Mycomya (Mycomya) cinerascens

Taxon classificationAnimaliaDipteraMycetophilidae

(Macquart, 1826)

4895B3D3-A7C8-5875-BAE3-1E05BAB923CA

###### Material.

1♂, SJ-8. Total: 1♂.

###### Distribution in Georgia.

Samtskhe-Javakheti.

###### General distribution.

Holarctic, extending to the Oriental region.

##### 39. Mycomya (Mycomya) flavicollis

Taxon classificationAnimaliaDipteraMycetophilidae

(Zetterstedt, 1852)

8C34CE3C-2248-5CBA-A57D-E38838550EF4

###### Material.

6♂♂, A-5; 1♂, A-7; 4♂♂, I-6; 2♂♂, SJ-3; 29♂♂, SJ-4; 1♂, SJ-9; 1♂, MM-7; 2♂♂, MM-14; 1♂, K-6. Total: 47♂♂.

###### Distribution in Georgia.

Adjara, Imereti, Samtskhe-Javakheti, Mtskhetha-Mthianethi, Kakheti.

###### General distribution.

Western Palaearctic.

###### Remarks.

In Transcaucasia recorded from Azerbaijan (Zaitzev 1994).

##### 40. Mycomya (Mycomya) griseovittata

Taxon classificationAnimaliaDipteraMycetophilidae

(Zetterstedt, 1852)

61EA199A-500A-50EF-A2CE-B6E8DA2FAB5F

###### Material.

1♂, SZS-3 (ZFMK). Total: 1♂.

###### Distribution in Georgia.

Samegrelo-Zemo Svanethi.

###### General distribution.

Holarctic.

##### 41. Mycomya (Mycomya) marginata

Taxon classificationAnimaliaDipteraMycetophilidae

(Meigen, 1818)

25281D7A-AC5B-56FE-B95A-D5D917668644

Fig. 9I

###### Material.

3♂♂, I-6; 1♂, I-17; 1♂, SJ-3; 2♂♂, MM-8; 1♂, MM-12. Total: 8♂♂.

###### Distribution in Georgia.

Imereti, Samtskhe-Javakheti, Mtskhetha-Mthianethi.

###### General distribution.

Palaearctic.

##### 42. Mycomya (Mycomya) occultans

Taxon classificationAnimaliaDipteraMycetophilidae

(Winnertz, 1863)

3DD22F87-1AD5-5CF6-B3D3-9F8D60BF3F9A

###### Material.

1♂, SJ-4. Total: 1♂.

###### Distribution in Georgia.

Samtskhe-Javakheti.

###### General distribution.

Palaearctic, extending to the Oriental region.

##### 43. Mycomya (Mycomya) tenuis

Taxon classificationAnimaliaDipteraMycetophilidae

(Walker, 1856)

FF7DE2A9-8F7C-5B38-932A-8D557EE1C859

###### Material.

1♂, A-7; 1♂, SJ-4. Total: 2♂♂.

###### Distribution in Georgia.

Adjara, Samtskhe-Javakheti.

###### General distribution.

Palaearctic.

##### 44. Mycomya (Mycomya) tridens

Taxon classificationAnimaliaDipteraMycetophilidae

(Lundström, 1911)

D682E1BD-A518-560A-9905-F5C975A5ACBD

###### Material.

1♂, SZS-4 (ZFMK). Total: 1♂.

###### Distribution in Georgia.

Samegrelo-Zemo Svanethi.

###### General distribution.

Europe.

##### 45. Mycomya (Mycomya) tumida

Taxon classificationAnimaliaDipteraMycetophilidae

(Winnertz, 1863)

2D79CC59-9BE6-55AF-94A5-22E3279E918C

###### Material.

1♂, SZS-4 (ZFMK); 1♂, I-11. Total: 2♂♂.

###### Distribution in Georgia.

Samegrelo-Zemo Svanethi, Imereti.

###### General distribution.

Palaearctic.

###### Remarks.

In Transcaucasia recorded from Azerbaijan (Zaitzev 1994).

##### 46. Mycomya (Mycomya) winnertzi

Taxon classificationAnimaliaDipteraMycetophilidae

(Dziedzicki, 1885)

98985B94-602E-51D3-BD00-92B2B19634B9

###### Material.

1♂, SK-1; 1♂, SJ-8. Total: 2♂♂.

###### Distribution in Georgia.

Shida Kartli, Samtskhe-Javakheti.

###### General distribution.

Palaearctic, extending to the Oriental region.

##### 47. Mycomya (Mycomyopsis) affinis

Taxon classificationAnimaliaDipteraMycetophilidae

(Staeger, 1840)

495D18DF-70D2-56A7-90FC-74782D2A95C3

###### Material.

3♂♂, K-5; 1♂, K-6. Total: 4♂♂.

###### Distribution in Georgia.

Kakheti.

###### General distribution.

Palaearctic.

##### 48. Mycomya (Mycomyopsis) trilineata

Taxon classificationAnimaliaDipteraMycetophilidae

(Zetterstedt, 1838)

0DBC55C6-A12C-523A-8FF8-0E4C93863828

###### Material.

5♂♂, K-6. Total: 5♂♂.

###### Distribution in Georgia.

Kakheti.

###### General distribution.

Palaearctic.

##### 49. Neoempheria brevilineata

Taxon classificationAnimaliaDipteraMycetophilidae

Okada, 1939

DF25961E-A463-5F46-B932-AE0FB8F592EC

Figs 9H , 10A–F

###### Material.

1♂, A-7; 2♂♂, I-6. Total: 3♂♂.

###### Distribution in Georgia.

Adjara, Imereti.

###### General distribution.

Palaearctic.

###### Remarks.

**Figure 10.**
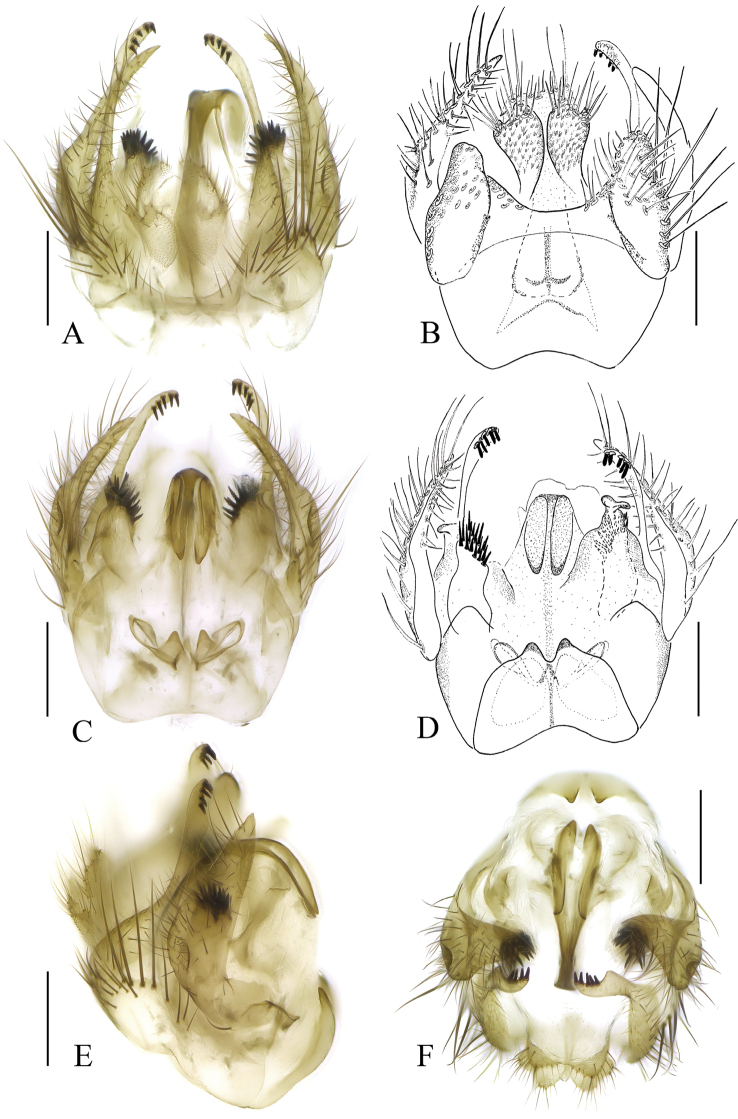
*Neoempheria brevilineata* Okada, 1939, male terminalia **A, B**, dorsal view **C, D** ventral view **E** lateral view **F** posterior view. Scale basr: 0.2 mm.

The species description from Hokkaido (Japan) by Okada was supplemented by a black and white figure of the general habitus including wing venation and pattern (Okada 1939: plate XVI, fig. 3). The Georgian material was compared to that from Japan (1 ♂, JAPAN, Honshu, Ishikawa Perfecture, Kanazawa City, Kakuma Campus, window trap, 14.vii-21.vii.2006, Indah, T. leg.; Kjærandsen J. det., TSZD-JKJ-111335) and the small differences in male terminalia are considered to be within intraspecific variation. Figures of the male terminalia (Fig. 10A–F) are provided for the first time for the species.

##### 50. Neoempheria striata

Taxon classificationAnimaliaDipteraMycetophilidae

(Meigen, 1818)

DB8D5FAC-9D5B-5039-9277-3B1A2E399ECF

Fig. 9G

###### Material.

1♀, I-6; 1♂ 1♀, I-14. Total: 1♂ 2♀♀.

###### Distribution in Georgia.

Imereti.

###### General distribution.

Palaearctic.

#### Subfamily Sciophilinae

##### 51. Acnemia amoena

Taxon classificationAnimaliaDipteraMycetophilidae

Winnertz, 1863

D29AA442-C278-5CC1-89B2-E191581F4D13

###### Material.

1♂, I-6. Total: 1♂.

###### Distribution in Georgia.

Imereti.

###### General distribution.

Palaearctic.

##### 52. Acnemia hyrcanica

Taxon classificationAnimaliaDipteraMycetophilidae

Zaitzev, 1984

5789E4C1-C0D8-570E-9E8A-E845F3F1DF04

###### Material.

1♂, SZS-3 (ZFMK); 1♂, I-6. Total: 2♂♂.

###### Distribution in Georgia.

Samegrelo-Zemo Svanethi, Imereti.

###### General distribution.

Caucasia.

###### Remarks.

Recorded earlier from North Caucasus and Azerbaijan (Zaitzev 1994).

##### 53. Acnemia nitidicollis

Taxon classificationAnimaliaDipteraMycetophilidae

(Meigen, 1818)

17ED99F2-7CD9-5C55-BDC4-214A370D0AEA

Fig. 9C

###### Material.

4♂♂, SZS-3 (2♂♂ ZFMK, 1♂ IUTG, 1♂ IZBE); 2♂♂, A-3; 2♂♂, A-7; 3♂♂ 1♀, K-4; 1♂, K-5; 1♂, K-6. Total: 13♂♂ 1♀.

###### Distribution in Georgia.

Samegrelo-Zemo Svanethi, Adjara, Kakheti.

###### General distribution.

Palaearctic.

##### 54. Allocotocera pulchella

Taxon classificationAnimaliaDipteraMycetophilidae

(Curtis, 1837)

7531BC30-B90C-53EB-81C4-7E94FA870802

Fig. 11H

###### Material.

1♂, SJ-2 (ZFMK). Total: 1♂.

###### Distribution in Georgia.

Samtskhe-Javakheti.

###### General distribution.

Holarctic.

**Figure 11.**
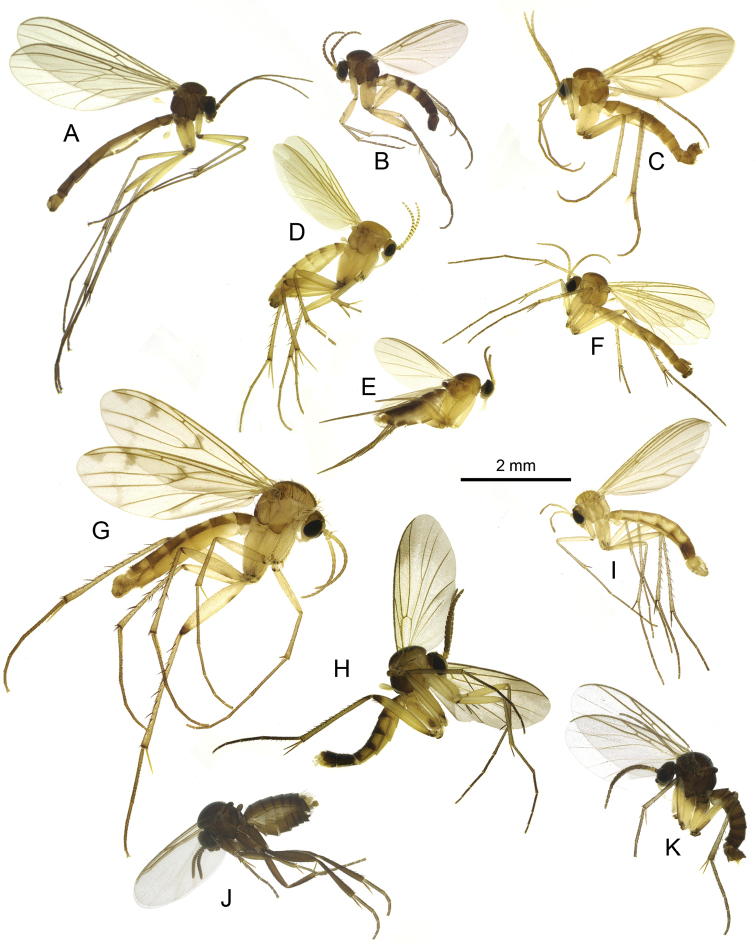
Habitus of Georgian fungus gnats of the family Mycetophilidae**A***Boletina trivittata* (Meigen, 1818) **B***Synapha fasciata* Meigen, 1818 **C***Grzegorzekia collaris* (Meigen, 1818) **D***Clastobasis loici* Chandler, 2001 **E***Manota unifurcata* Lundström, 1913 **F***Megalopelma nigroclavatum* (Strobl, 1910) **G***Leia winthemii* Lehmann, 1822 **H***Allocotocera pulchella* (Curtis, 1837) **I***Coelosia flava* (Staeger, 1840) **J***Novakia scatopsiformis* Strobl, 1893 **K***Ectrepesthoneura hirta* (Winnertz, 1846).

##### 55. Anaclileia adjarica

Taxon classificationAnimaliaDipteraMycetophilidae

Kurina, 2018

68B53D2A-E400-515D-BE94-128D8279B7B7

###### Georgian source.

Kurina 2018: 156 (figs 2–5).

###### Type material.

1♂, A-1 (holotype); 3♂♂, A-1 (paratypes); 3♂♂ 3♀♀, A-3 (paratypes); 1♂, A-7 (paratype). Total: 8♂♂ 3♀♀.

###### Distribution in Georgia.

Adjara.

###### General distribution.

Georgia.

###### Remarks.

The species was recently described from material collected from Mtirala and Kintrishi National Parks in Adjara (Kurina 2018)

##### 56. Azana (Azana) anomala

Taxon classificationAnimaliaDipteraMycetophilidae

(Staeger, 1840)

D08B9435-C761-558A-AD7B-DB940A759FB8

Fig. 9E

###### Material.

1♀, SJ-3. Total: 1♀.

###### Distribution in Georgia.

Samtskhe-Javakheti.

###### General distribution.

Europe.

##### 57. Azana (Jugazana) nigricoxa

Taxon classificationAnimaliaDipteraMycetophilidae

Strobl, 1898

C384700E-8732-5365-BD58-A2FC230E3D73

###### Material.

1♂, I-11. Total: 1♂.

###### Distribution in Georgia.

Imereti.

###### General distribution.

Europe.

##### 58. Megalopelma nigroclavatum

Taxon classificationAnimaliaDipteraMycetophilidae

(Strobl, 1910)

0DF81BD0-A92E-5D8A-A2E0-6C46C6717981

Fig. 11F

###### Material.

2♂♂, I-6. Total: 2♂♂.

###### Distribution in Georgia.

Imereti.

###### General distribution.

Holarctic.

##### 59. Monoclona rufilatera

Taxon classificationAnimaliaDipteraMycetophilidae

(Walker, 1837)

225E1194-548C-54A0-9A92-AA678EFB7B94

Fig. 9D

###### Material.

3♂♂, I-6; 1♂, SK-1. Total: 4♂♂.

###### Distribution in Georgia.

Imereti, Shida Kartli.

###### General distribution.

Holarctic.

###### Remarks.

In Transcaucasia recorded from Azerbaijan (Zaitzev 1994).

##### 60. Neuratelia caucasica

Taxon classificationAnimaliaDipteraMycetophilidae

Zaitzev, 1994

A290BF96-DECB-5791-A4AC-E60754CCEF34

###### Georgian source.

**Figure 12.**
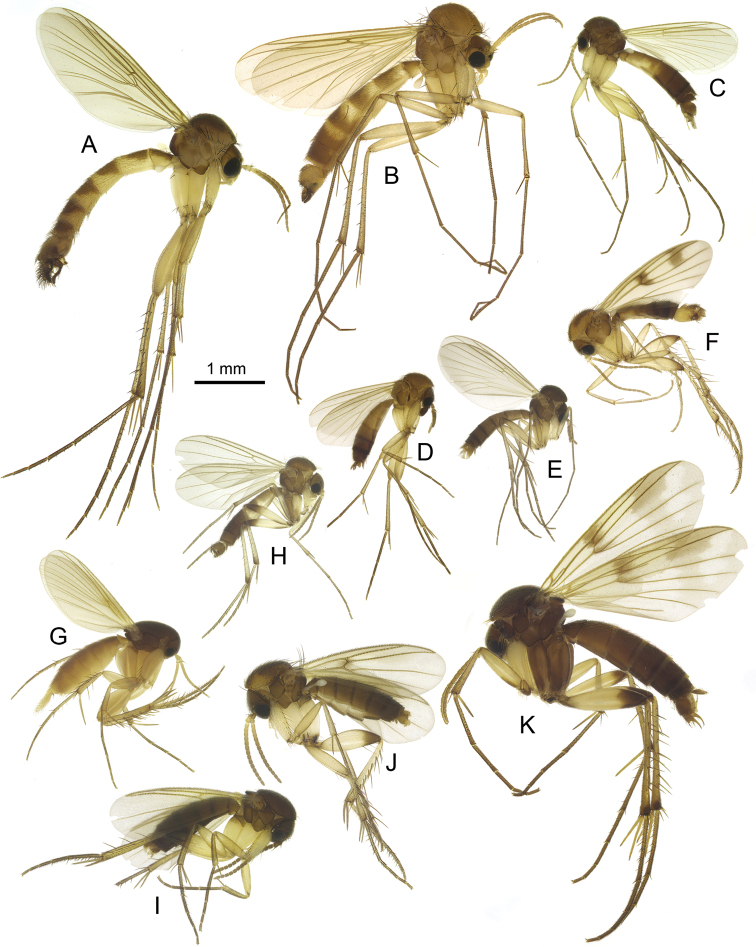
Habitus of Georgian fungus gnats of the family Mycetophilidae**A***Allodiopsis domestica* (Meigen, 1830) **B***Rymosia affinis* Winnertz, 1863 **C***Brevicornu griseicolle* (Staeger, 1840) **D***Cordyla fissa* Edwards, 1925 **E***Anatella longisetosa* Dziedzicki, 1923 **F***Mycetophila magnicauda* Strobl, 1895 **G***Epicypta scatophora* (Perris, 1849) **H***Phronia tenuis* Winnertz, 1863 **I***Sceptonia tenuis* Edwards, 1925 **J***Zygomyia humeralis* (Wiedemann, 1817) **K***Dynatosoma reciprocum* (Walker, 1848).

Kurina et al. 2015: 116 (figs 11, 12, 16).

###### Material.

1♀, SZS-3 (IZBE); 2♀♀, SZS-4 (1♀ ZFMK, 1♀ IUTG); 3♂♂ 4♀♀, A-3; 1♂ 2♀♀, A-7; 2♂♂ 10♀♀, I-6; 1♂ 1♀, I-9; 2♂♂ 2♀♀, SK-1; 1♂, SJ-1 (IZBE); 4♂♂ 1♀, SJ-2 (2♂♂ ZFMK, 1♂ 1♀ IUTG, 1♂ IZBE); 2♂♂ 1♀, SJ-3; 1♂, KK-1. Total: 17♂♂ 24♀♀.

###### Distribution in Georgia.

Samegrelo-Zemo Svanethi, Adjara, Imereti, Shida Kartli, Samtskhe-Javakheti, Kvemo Kartli.

###### General distribution.

Caucasia: Russia (Krasnodarskiy region), Georgia.

##### 61. Phthinia hyrcanica

Taxon classificationAnimaliaDipteraMycetophilidae

Zaitzev, 1984

22356499-8476-5106-907F-83E6C85D0A45

Fig. 9F

###### Material.

1♂, MM-8; 1♂, K-4. Total: 2♂♂.

###### Distribution in Georgia.

Mtskhetha-Mthianethi, Kakheti.

###### General distribution.

Caucasia.

###### Remarks.

Known only from type locality in Azerbaijan (Zaitzev 1994).

##### 62. Polylepta zonata

Taxon classificationAnimaliaDipteraMycetophilidae

Zetterstedt, 1852

1FE13894-88B6-5E0C-B321-27E001B5ACEC

###### Material.

1♂, A-5. Total: 1♂.

###### Distribution in Georgia.

Adjara.

###### General distribution.

Europe, with scattered distribution (Kurina 2003, Chandler 2013).

##### 63. Sciophila fenestella

Taxon classificationAnimaliaDipteraMycetophilidae

Curtis, 1837

CF4627B6-5F85-5D2E-8D9D-20C077CAE4CC

###### Material.

1♂, I-6. Total: 1♂.

###### Distribution in Georgia.

Imereti.

###### General distribution.

Holarctic.

##### 64. Sciophila georgei sp. nov.

Taxon classificationAnimaliaDipteraMycetophilidae

6EE3AE2C-3471-5F13-BA0B-7E5D8502FBDC

###### Material.

See in species description above.

###### Distribution in Georgia.

Kakheti.

###### General distribution.

Georgia.

##### 65. Sciophila nitens

Taxon classificationAnimaliaDipteraMycetophilidae

(Winnertz, 1863)

3134B590-2BCE-59CA-9FFC-A0CE93C7E6C0

###### Material.

1♂, SZS-3 (ZFMK); 1♂, SJ-1 (IZBE); 1♂, SJ-2 (IUTG). Total: 3♂♂.

###### Distribution in Georgia.

Samegrelo-Zemo Svanethi, Samtskhe-Javakheti.

###### General distribution.

Holarctic.

###### Remarks.

In Europe recorded from mountain areas (Kurina 2004, 2008b).

##### 66. Sciophila thoracica

Taxon classificationAnimaliaDipteraMycetophilidae

Staeger, 1840

4FDE1722-BCB9-507A-8F5B-6F7C70AAD159

###### Material.

1♂, SZS-3 (IZBE); 1♂, SJ-2 (ZFMK). Total: 2♂♂.

###### Distribution in Georgia.

Samegrelo-Zemo Svanethi, Samtskhe-Javakheti.

###### General distribution.

Holarctic.

##### 67. Syntemna morosa

Taxon classificationAnimaliaDipteraMycetophilidae

Winnertz, 1863

5D3F2934-FE2A-5306-8121-4D0272A67058

###### Material.

1♂, I-6; 1♂, I-9. Total: 2♂♂.

###### Distribution in Georgia.

Imereti.

###### General distribution.

Europe.

#### Subfamily Gnoristinae

##### 68. Apolephthisa subincana

Taxon classificationAnimaliaDipteraMycetophilidae

(Curtis, 1837)

BF30F735-4D5E-55EA-9F95-A911BDF665A9

###### Material.

1♂, I-6. Total: 1♂.

###### Distribution in Georgia.

Imereti.

###### General distribution.

Western Palaearctic.

##### 69. Boletina borealis

Taxon classificationAnimaliaDipteraMycetophilidae

Zetterstedt, 1852

633FA627-564F-5F1A-8B20-8A2A9D41B542

###### Material.

1♂, SZS-2; 1♂, SZS-4 (ZFMK). Total: 2♂♂.

###### Distribution in Georgia.

Samegrelo-Zemo Svanethi.

###### General distribution.

Palaearctic.

##### 70. Boletina digitata

Taxon classificationAnimaliaDipteraMycetophilidae

Lundström, 1914

63508AE0-B752-5523-80C9-406BAB6B3B98

###### Material.

4♂♂, SZS-4 (2♂♂ ZFMK, 1♂ IUTG, 1♂ IZBE). Total: 4♂♂.

###### Distribution in Georgia.

Samegrelo-Zemo Svanethi.

###### General distribution.

Western Palaearctic.

##### 71. Boletina dubia

Taxon classificationAnimaliaDipteraMycetophilidae

(Meigen, 1804)

48EC3D3A-5480-50A9-B8A9-4C5B5F93DCD9

###### Material.

5♂♂, A-1; 3♂♂, A-3. Total: 8♂♂.

###### Distribution in Georgia.

Adjara.

###### General distribution.

Europe.

###### Remarks.

The Georgian specimens have the ventral lobe of the gonostylus with a blunt small spine apically that is absent in studied specimens from Estonia and Sweden as well as in published figures (e.g. Landrock 1927, Hutson et al. 1980, Zaitzev 1994). Otherwise, the male terminalia including aedeagal complex do not have any substantial differences. Therefore, the Georgian material is considered to be conspecific pending further, more thorough study including that based on DNA sequencing.

##### 72. Boletina gripha

Taxon classificationAnimaliaDipteraMycetophilidae

Dziedzicki, 1885

5FA5CCF4-3BCF-50EB-84F6-82CBE46B2E42

###### Material.

2♂♂, SZS-4 (1♂ ZFMK, 1♂ IZBE). Total: 2♂♂.

###### Distribution in Georgia.

Samegrelo-Zemo Svanethi.

###### General distribution.

Palaearctic.

##### 73. Boletina moravica

Taxon classificationAnimaliaDipteraMycetophilidae

Landrock, 1912

E43EFD86-E6C1-5DEF-A268-CE6AF48E90C1

###### Material.

1♂, SZS-4 (ZFMK). Total: 1♂.

###### Distribution in Georgia.

Samegrelo-Zemo Svanethi.

###### General distribution.

Europe.

##### 74. Boletina nitida

Taxon classificationAnimaliaDipteraMycetophilidae

Grzegorzek, 1885

AC9A0035-CEA8-5B62-8A9D-B7D556D80C06

###### Material.

10♂♂, SZS-3 (4♂♂ ZFMK, 4 ♂♂ IUTG, 2 ♂♂ IZBE); 1♂, SJ-1 (IZBE); 1♂, K-6. Total: 12♂♂.

###### Distribution in Georgia.

Samegrelo-Zemo Svanethi, Samtskhe-Javakheti, Kakheti.

###### General distribution.

Palaearctic.

##### 75. Boletina sciarina

Taxon classificationAnimaliaDipteraMycetophilidae

Staeger, 1840

487D5158-52C5-5676-B22D-9E5F6594FF91

###### Material.

3♂♂, SZS-4 (1♂ ZFMK, 1♂ IUTG, 1♂ IZBE). Total: 3♂♂.

###### Distribution in Georgia.

Samegrelo-Zemo Svanethi.

###### General distribution.

Holarctic.

##### 76. Boletina trivittata

Taxon classificationAnimaliaDipteraMycetophilidae

(Meigen, 1818)

63D861C9-92F3-5D36-A993-E389101C3CB9

Fig. 11A

###### Material.

4♂♂, SJ-8; 5♂♂, SJ-9. Total: 9♂♂.

###### Distribution in Georgia.

Samtskhe-Javakheti.

###### General distribution.

Palaearctic.

##### 77. Coelosia flava

Taxon classificationAnimaliaDipteraMycetophilidae

(Staeger, 1840)

64F04AF0-AEC2-5E3B-9C90-CEC84EC8EE90

Fig. 11I

###### Georgian source.

Thormann et al. 2019: 279 (from Mtskhetha-Mthianethi).

###### Material.

1♂, SZS-3 (ZFMK); 8♂♂ 1♀, KK-1. Total: 9♂♂ 1♀.

###### Distribution in Georgia.

Samegrelo-Zemo Svanethi, Mtskhetha-Mthianethi, Kvemo Kartli.

###### General distribution.

Europe.

##### 78. Docosia gilvipes

Taxon classificationAnimaliaDipteraMycetophilidae

(Haliday in Walker, 1856)

E27A7B78-1470-5913-B3E0-83E3673B132C

###### Georgian source.

Ševčík et al. 2020: 21

###### Material.

1♂, SZS-3 (ZFMK); 1♀, I-6. Total: 1♂ 1♀.

###### Distribution in Georgia.

Samegrelo-Zemo Svanethi, Imereti.

###### General distribution.

Palaearctic.

##### 79. Docosia flavicoxa

Taxon classificationAnimaliaDipteraMycetophilidae

Strobl, 1900

12CE84F6-DDE7-598E-A151-C569B048236E

###### Georgian source.

Ševčík et al. 2020: 21

###### Material.

3♂♂, K-5. Total: 3♂♂.

###### Distribution in Georgia.

Kakheti.

###### General distribution.

Europe.

##### 80. Docosia moravica

Taxon classificationAnimaliaDipteraMycetophilidae

Landrock, 1916

434C7BD2-A668-5817-B3B3-5A59EE2C7B22

###### Georgian source.

Ševčík et al. 2020: 23.

###### Material.

1♂, SJ-2 (ZFMK). Total: 1♂.

###### Distribution in Georgia.

Samtskhe-Javakheti.

###### General distribution.

Palaearctic.

##### 81. Docosia pannonica

Taxon classificationAnimaliaDipteraMycetophilidae

Laštovka & Ševčík, 2006

DD551539-E4B9-53FB-895C-60C83C1FFBFC

###### Georgian source.

Ševčík et al. 2020: 23

###### Material.

1♂, MM-3. Total: 1♂.

###### Distribution in Georgia.

Mtskhetha-Mthianethi.

###### General distribution.

Europe.

###### Remarks.

Known from Central Europe (Laštovka and Ševčík 2006).

##### 82. Docosia svanetica

Taxon classificationAnimaliaDipteraMycetophilidae

Kurina in Ševčík et al. 2020

7A04D1D5-967F-5A14-80FB-B6340A401C88

###### Georgian source.

Ševčík et al. 2020: 17.

###### Material.

1♂, SZS-4 (holotype, ZFMK); 5♂♂, SZS-4; 2♂♂, SZS-3; 1♂, SJ-1. Total: 9♂♂ (see Ševčík et al. 2020 for depository of paratypes).

###### Distribution in Georgia.

Samegrelo-Zemo Svanethi, Samtskhe-Javakheti.

###### General distribution.

Georgia.

##### 83. Ectrepesthoneura hirta

Taxon classificationAnimaliaDipteraMycetophilidae

(Winnertz, 1846)

712B5F01-42A6-54CB-8030-F90A70CD27C7

Fig. 11K

###### Material.

1♂, SJ-2 (ZFMK); 1♂, K-6. Total: 2♂♂.

###### Distribution in Georgia.

Samtskhe-Javakheti, Kakheti.

###### General distribution.

Europe.

##### 84. Grzegorzekia collaris

Taxon classificationAnimaliaDipteraMycetophilidae

(Meigen, 1818)

C73BA377-2968-5916-A3E4-A6752FDDF1A9

Fig. 11C

###### Georgian source.

Thormann et al. 2019: 279 (from Mtskhetha-Mthianethi).

###### Material.

2♂♂, SZS-3 (1♂ ZFMK, 1♂ IZBE); 1♂, I-10; 1♂, SK-1. Total: 4♂♂.

###### Distribution in Georgia.

Samegrelo-Zemo Svanethi, Imereti, Shida Kartli, Mtskhetha-Mthianethi.

###### General distribution.

Palaearctic.

##### 85. Lusitanoneura chandleri

Taxon classificationAnimaliaDipteraMycetophilidae

(Caspers, 1991)

30D79A86-22F0-59D4-B417-D4ECBA9A9221

###### Material.

1♂, SZS-3 (ZFMK); 2♂♂, I-6. Total: 3♂♂.

###### Distribution in Georgia.

Samegrelo-Zemo Svanethi, Imereti.

###### General distribution.

Europe.

###### Remarks.

Known only from Grete and Cyprus (Caspers 1991, Chandler et al. 2006, Ribeiro and Chandler 2007).

##### 86. Palaeodocosia vittata

Taxon classificationAnimaliaDipteraMycetophilidae

(Coquillett, 1901)

949C8852-9A16-5FDC-BEE7-2ECAC1C4987D

###### Material.

1♂, A-1; 1♂, I-6. Total: 2♂♂.

###### Distribution in Georgia.

Adjara, Imereti.

###### General distribution.

Holarctic.

##### 87. Synapha fasciata

Taxon classificationAnimaliaDipteraMycetophilidae

Meigen, 1818

D9B33DD0-CD21-536A-A4B5-73B1EE00A3C3

Fig. 11B

###### Material.

104♂♂, A-1; 54♂♂ 5♀♀, A-2; 70♂♂, A-3; 21♂♂ 2♀♀, A-4; 6♂♂ 4♀♀, A-5; 3♂♂, A-6; 173♂♂ 64♀♀, A-7; 14♂♂, I-1; 7♂♂, I-3 (18.v–1.vi.2013); 1♂, I-4; 138♂♂ 7♀♀, I-6; 25♂♂, I-7; 2♂♂ 3♀♀, I-9; 1♀, I-10; 8♂♂, I-11; 15♂♂, I-13; 2♂♂, I-16; 37♂♂, I-17; 1♂, MM-9. Total: 683♂♂ 86♀♀.

###### Distribution in Georgia.

Adjara, Imereti, Mtskhetha-Mthianethi.

###### General distribution.

Palaearctic.

#### Subfamily Leiinae

##### 88. Clastobasis alternans

Taxon classificationAnimaliaDipteraMycetophilidae

(Winnertz, 1863)

CA169BC7-C957-55D8-A597-66A52AB46E0A

###### Material.

6♂♂ 2♀♀, SZS-3 (2♂♂ 1♀ ZFMK, 2♂♂ 1♀ IUTG, 2♂♂ IZBE); 1♂, K-4; 1♂, K-5. Total: 8♂♂ 2♀♀.

###### Distribution in Georgia.

Samegrelo-Zemo Svanethi, Kakheti.

###### General distribution.

Palaearctic.

##### 89. Clastobasis loici

Taxon classificationAnimaliaDipteraMycetophilidae

Chandler, 2001

FC22FF1A-8D9A-5C36-99D5-BC9D9FD82C0B

Fig. 11D

###### Material.

3♂♂, K-4; 22♂♂, K-5. Total: 25♂♂.

###### Distribution in Georgia.

Kakheti.

###### General distribution.

Palaearctic.

###### Remarks.

This very rare species was until recently recorded only from Channel Islands and Central Europe but Kurina (2020b) found it also from Japan. The record from Georgia suggests a wider distribution in the Palearctic region.

##### 90. Greenomyia mongolica

Taxon classificationAnimaliaDipteraMycetophilidae

Laštovka & Matile, 1974

BFBFEEE0-1BEF-5B3F-968D-173454C76886

###### Material.

1♂, SJ-5. Total: 1♂.

###### Distribution in Georgia.

Samtskhe-Javakheti.

###### General distribution.

Palaearctic.

###### Remarks.

A widely distributed Palaearctic species that has expanded its range in Europe during recent decades and is locally common also in anthropogenic environments (Kurina et al. 2011, *pers. observations*).

##### 91. Leia bimaculata

Taxon classificationAnimaliaDipteraMycetophilidae

(Meigen, 1804)

B10E0BD9-4088-50F6-B9F8-82D15A116032

###### Material.

1♂, SZS-3 (IZBE); 1♂, SK-1; 1♂, SJ-2 (ZFMK); 1♂, SJ-9; 1♂, MM-7. Total: 5♂♂.

###### Distribution in Georgia.

Samegrelo-Zemo Svanethi, Shida Kartli, Samtskhe-Javakheti, Mtskhetha-Mthianethi.

###### General distribution.

Palaearctic.

###### Remarks.

Aedeagal complex of Georgian specimens is similar to that figured by Polevoi and Salmela (2016: fig. 7G) and gonostylus has a clear dorsal projection (Polevoi and Salmela 2016: fig. 7I, J).

##### 92. Leia cylindrica

Taxon classificationAnimaliaDipteraMycetophilidae

(Winnertz, 1863)

1C32D3A3-0325-5C23-98AB-752DBF5317A0

###### Material.

1♂ 1♀, I-6; 1♂, K-6. Total: 2♂♂ 1♀.

###### Distribution in Georgia.

Imereti, Kakheti.

###### General distribution.

Western Palaearctic.

##### 93. Leia katae sp. nov.

Taxon classificationAnimaliaDipteraMycetophilidae

237FE660-B57B-5DEB-91F9-5495E0480CE1

###### Material.

See in species description above.

###### Distribution in Georgia.

Samegrelo-Zemo-Svanethi, Shida-Kartli.

###### General distribution.

Georgia.

##### 94. Leia piffardi

Taxon classificationAnimaliaDipteraMycetophilidae

Edwards, 1925

F846E2BF-2A57-5FAD-AD1B-E67C802CA9C7

###### Material.

1♂, I-6. Total: 1♂.

###### Distribution in Georgia.

Imereti.

###### General distribution.

Europe, with scattered distribution.

##### 95. Leia winthemii

Taxon classificationAnimaliaDipteraMycetophilidae

Lehmann, 1822

BDED0737-CDA0-5D3B-A7E3-F00BA38C7595

Fig. 11G

###### Material.

3♂♂, I-10; 1♂, SJ-8. Total: 4♂♂.

###### Distribution in Georgia.

Imereti, Samtskhe-Javakheti.

###### General distribution.

Holarctic, extending to the Oriental region.

##### 96. Novakia scatopsiformis

Taxon classificationAnimaliaDipteraMycetophilidae

Strobl, 1893

CC3BD89D-C0FC-5ACB-B0F2-998DB7ACA30A

Fig. 11J

###### Material.

1♂ 1♀, SJ-2 (1♀ ZFMK, 1♂ IZBE); 1♂, K-4; 1♀, K-5. Total: 2♂♂ 2♀♀.

###### Distribution in Georgia.

Samtskhe-Javakheti, Kakheti.

###### General distribution.

Western Palaearctic.

###### Remarks.

According to the recent molecular study by Kaspřák et al. (2019: Fig. 1), the genus *Novakia* Strobl, 1893 apparently belongs to the subfamily Gnoristinae. However, as the authors did not have further discussion about this relationship, I follow the current classification in Fungus Gnats Online (http://www.sciaroidea.info/).

#### Subfamily Manotinae

##### 97. Manota unifurcata

Taxon classificationAnimaliaDipteraMycetophilidae

Lundström, 1913

2229D9F3-DFB4-5203-B11C-71248796DFC2

Fig. 11E

###### Material.

1♂, I-6. Total: 1♂.

###### Distribution in Georgia.

Imereti.

###### General distribution.

Europe.

###### Remarks.

A rare species, recorded from Central and Northern Europe with the south-eastern record on the Crimean Peninsula (Jaschhof et al. 2011). The current record from Georgia indicates a wider distribution in the Western Palaearctic.

#### Subfamily Mycetophilinae

**Tribe Exechiini**

##### 98. Allodia lugens

Taxon classificationAnimaliaDipteraMycetophilidae

(Wiedemann, 1817)

A2A998A9-4689-5EFA-A56A-A19241D0FFD4

###### Material.

4♂♂, SZS-4 (2♂♂ ZFMK, 1♂ IUTG, 1♂ IZBE); 7♂♂, SJ-3; 1♂, SJ-8; 1♂, MM-2; 1♂, MM-11; 1♂, MM-12. Total: 15♂♂.

###### Distribution in Georgia.

Samegrelo-Zemo Svanethi, Samtskhe-Javakheti, Mtskhetha-Mthianethi.

###### General distribution.

Holarctic.

###### Remarks.

Listed to occur in Transcaucasia without further details (Zaitzev 2003).

##### 99. Allodia ornaticollis

Taxon classificationAnimaliaDipteraMycetophilidae

(Meigen, 1818)

A3D5387A-CEDA-5A08-AACD-FA64096B4E89

###### Material.

1♂, SJ-9; 1♂, MM-12; 1♂, MM-14; 1♂, K-1. Total: 4♂♂.

###### Distribution in Georgia.

Samtskhe-Javakheti, Mtskhetha-Mthianethi, Kakheti.

###### General distribution.

Holarctic.

###### Remarks.

In Transcaucasia recorded from Azerbaijan (Zaitzev 2003).

##### 100. Allodia truncata

Taxon classificationAnimaliaDipteraMycetophilidae

Edwards, 1921

0E9B6530-E974-52E7-908C-6CDBDCEEE3F4

###### Material.

1♂, SJ-8. Total: 1♂.

###### Distribution in Georgia.

Samtskhe-Javakheti.

###### General distribution.

Holarctic.

##### 101. Allodiopsis domestica

Taxon classificationAnimaliaDipteraMycetophilidae

(Meigen, 1830)

23C27DB0-0CDB-5FD4-9F28-F0435E5D2E4A

Fig. 12A

###### Material.

1♂, A-7; 2♂♂, MM-2; 1♂, MM-8. Total: 4♂♂.

###### Distribution in Georgia.

Adjara, Mtskhetha-Mthianethi.

###### General distribution.

Holarctic.

###### Remarks.

Listed to occur in Transcaucasia without further details (Zaitzev 2003).

##### 102. Allodiopsis korolevi

Taxon classificationAnimaliaDipteraMycetophilidae

Zaitzev, 1982

D06F43DF-EE77-52B0-8841-08173AC2FDEF

###### Material.

1♂, SZS-4 (ZFMK); 1♂, SJ-9. Total: 2♂♂.

###### Distribution in Georgia.

Samegrelo-Zemo Svanethi, Samtskhe-Javakheti.

###### General distribution.

Palaearctic.

##### 103. Allodiopsis rustica

Taxon classificationAnimaliaDipteraMycetophilidae

(Edwards, 1941)

094C77D4-DCE1-5795-9268-B233EBDAA665

###### Material.

2♂♂, SJ-8; 1♂, MM-12. Total: 3♂♂.

###### Distribution in Georgia.

Samtskhe-Javakheti, Mtskhetha-Mthianethi.

###### General distribution.

Palaearctic.

##### 104. Anatella longisetosa

Taxon classificationAnimaliaDipteraMycetophilidae

Dziedzicki, 1923

8DF71C57-33C3-5F69-A9CD-F7C591336DAD

Fig. 12E

###### Material.

1♂, SZS-3 (ZFMK); 3♂♂, SZS-4 (1♂ ZFMK, 1♂ IUTG, 1♂ IZBE). Total: 4♂♂.

###### Distribution in Georgia.

Samegrelo-Zemo Svanethi.

###### General distribution.

Europe.

##### 105. Anatella metae sp. nov.

Taxon classificationAnimaliaDipteraMycetophilidae

57815A80-72CE-5655-9360-BCDA7F716102

###### Material.

See in species description above.

###### Distribution in Georgia.

Mtskhetha-Mthianethi.

###### General distribution.

Georgia.

##### 106. Anatella simpatica

Taxon classificationAnimaliaDipteraMycetophilidae

Dziedzicki, 1923

EFDEF363-648C-5BBF-86AE-669676D0D3AB

###### Material.

1♂, MM-2; 1♂, MM-12. Total: 2♂♂.

###### Distribution in Georgia.

Mtskhetha-Mthianethi.

###### General distribution.

Holarctic.

##### 107. Brachycampta alternans

Taxon classificationAnimaliaDipteraMycetophilidae

(Zetterstedt, 1838)

AD712DBD-B0A1-5985-ACB7-7F565E61E8D6

###### Material.

1♂, MM-14. Total: 1♂.

###### Distribution in Georgia.

Mtskhetha-Mthianethi.

###### General distribution.

Holarctic.

##### 108. Brachycampta czernyi

Taxon classificationAnimaliaDipteraMycetophilidae

(Landrock, 1912)

B49B6A48-FAF6-5DA0-A101-EB77014367DB

###### Material.

1♂, MM-2. Total: 1♂.

###### Distribution in Georgia.

Mtskhetha-Mthianethi.

###### General distribution.

Holarctic.

##### 109. Brachycampta grata

Taxon classificationAnimaliaDipteraMycetophilidae

(Meigen, 1830)

8F4B8BFF-06B7-56B7-9B14-DFC2F0C9F72F

###### Material.

4♂♂, I-6; 1♂, I-10; 1♂, SJ-3; 1♂, MM-5; 1♂, MM-8. Total: 8♂♂.

###### Distribution in Georgia.

Imereti, Samtskhe-Javakheti, Mtskhetha-Mthianethi.

###### General distribution.

Palaearctic.

###### Remarks.

In Transcaucasia recorded from Azerbaijan (Zaitzev 2003).

##### 110. Brachycampta foliifera

Taxon classificationAnimaliaDipteraMycetophilidae

(Strobl, 1910)

42E51E02-4040-5C87-805E-0FE15C17B64F

###### Material.

1♂, MM-2. Total: 1♂.

###### Distribution in Georgia.

Mtskhetha-Mthianethi.

###### General distribution.

Holarctic.

###### Remarks.

In Transcaucasia recorded from Azerbaijan (Zaitzev 2003).

##### 111. Brachycampta neglecta

Taxon classificationAnimaliaDipteraMycetophilidae

Edwards, 1925

102425D3-7B65-57A3-B9D0-02DEF8F20A8E

###### Material.

1♂, I-6. Total: 1♂.

###### Distribution in Georgia.

Imereti.

###### General distribution.

Palaearctic.

##### 112. Brachycampta pistillata

Taxon classificationAnimaliaDipteraMycetophilidae

(Lundström, 1911)

BE4FEB63-41D6-5F74-A9F1-C99B13C4297B

###### Material.

5♂♂, I-6. Total: 5♂♂.

###### Distribution in Georgia.

Imereti.

###### General distribution.

Holarctic.

###### Remarks.

In Transcaucasia recorded from Azerbaijan (Zaitzev 2003).

##### 113. Brachycampta protenta

Taxon classificationAnimaliaDipteraMycetophilidae

Laštovka & Matile, 1974

118594DB-2746-5235-A04D-7486D1780615

###### Material.

1♂, SZS-4 (ZFMK). Total: 1♂.

###### Distribution in Georgia.

Samegrelo-Zemo Svanethi.

###### General distribution.

Holarctic.

##### 114. Brachycampta westerholti

Taxon classificationAnimaliaDipteraMycetophilidae

Caspers, 1980

94B86743-69E8-50AD-9E63-DFC653F36889

###### Material.

1♂, SJ-8. Total: 1♂.

###### Distribution in Georgia.

Samtskhe-Javakheti.

###### General distribution.

Western Palaearctic.

###### Remarks.

In Transcaucasia recorded from Azerbaijan (Zaitzev 2003).

##### 115. Brevicornu auriculatum

Taxon classificationAnimaliaDipteraMycetophilidae

(Edwards, 1925)

1888F37D-94BF-5C07-B033-6CFCEDE5AAA5

###### Material.

1♂, SZS-4 (ZFMK); 1♂, A-8. Total: 2♂♂.

###### Distribution in Georgia.

Samegrelo-Zemo Svanethi, Adjara.

###### General distribution.

Palaearctic.

##### 116. Brevicornu bellum

Taxon classificationAnimaliaDipteraMycetophilidae

(Johannsen, 1912)

94B62DE6-BD86-5E5A-A6E3-FCB214A0DE06

###### Material.

1♂, SZS-4 (ZFMK). Total: 1♂.

###### Distribution in Georgia.

Samegrelo-Zemo Svanethi.

###### General distribution.

Holarctic.

##### 117. Brevocornu fuscipenne

Taxon classificationAnimaliaDipteraMycetophilidae

(Staeger, 1840)

741831C8-147F-5443-8E44-CD19CF4F05EC

###### Material.

1♂, I-6; 1♂, SJ-8. Total: 2♂♂.

###### Distribution in Georgia.

Imereti, Samtskhe-Javakheti.

###### General distribution.

Holarctic.

##### 118. Brevicornu griseicolle

Taxon classificationAnimaliaDipteraMycetophilidae

(Staeger, 1840)

1D2704EF-F6CC-5F56-94AE-F9BE10D0516F

Fig. 12C

###### Material.

8♂♂, SZS-4 (3♂♂ ZFMK, 3♂♂ IUTG, 2♂♂ IZBE); 1♂, I-1; 2♂♂, I-6; 1♂, SK-1; 1♂, SJ-6; 4♂♂, SJ-7; 1♂, SJ-9; 1♂, MM-2; 1♂, MM-14; 1♂, K-1. Total: 21♂♂.

###### Distribution in Georgia.

Samegrelo-Zemo Svanethi, Imereti, Shida Kartli, Samtskhe-Javakheti, Mtskhetha-Mthianethi, Kakheti.

###### General distribution.

Palaearctic.

##### 119. Brevicornu intermedium

Taxon classificationAnimaliaDipteraMycetophilidae

(Santos Abreu, 1920)

91DF01BD-E92C-51D2-8E25-8AFA85835F84

###### Material.

1♂, I-11, 2♂♂, SJ-7; 2♂♂, SJ-8; 1♂, SJ-9; 1♂, MM-14. Total: 7♂♂.

###### Distribution in Georgia.

Imereti, Samtskhe-Javakheti, Mtskhetha-Mthianethi.

###### General distribution.

Western Palaearctic.

##### 120. Brevicornu proximum

Taxon classificationAnimaliaDipteraMycetophilidae

(Staeger, 1840)

3A698037-EB4B-5661-8460-9460DF2F6BF0

###### Material.

2♂♂, I-6; 1♂, I-11; 1♂, SJ-8; 1♂, SJ-9; 1♂, MM-5; 2♂♂, MM-12; 1♂, MM-13. Total: 9♂♂.

###### Distribution in Georgia.

Imereti, Samtskhe-Javakheti, Mtskhetha-Mthianethi.

###### General distribution.

Palaearctic.

###### Remarks.

In Transcaucasia recorded from Azerbaijan (Zaitzev 2003).

##### 121. Brevicornu sericoma

Taxon classificationAnimaliaDipteraMycetophilidae

(Meigen, 1830)

0AA820C9-1F8C-552B-82D9-03E41124CA31

###### Material.

1♂, SZS-3 (ZFMK); 1♂, SZS-4 (IZBE); 1♂, SJ-7; 4♂♂, SJ-8; 1♂, SJ-9; 3♂♂, KK-1; 1♂, MM-2; 1♂, MM-8; 1♂, MM-14; 1♂, K-2. Total: 15♂♂.

###### Distribution in Georgia.

Samegrelo-Zemo Svanethi, Samtskhe-Javakheti, Kvemo Kartli, Mtskhetha-Mthianethi.

###### General distribution.

Holarctic.

###### Remarks.

Listed to occur in Transcaucasia without further details (Zaitzev 2003).

##### 122. Cordyla brevicornis

Taxon classificationAnimaliaDipteraMycetophilidae

(Staeger, 1840)

0D62428F-A3A5-569D-9457-0F6C0C6E7DC1

###### Material.

1♂, SZS-3 (IZBE); 5♂♂, SZS-4 (3♂♂ ZFMK, 2♂♂ IUTG); 1♂, A-5; 1♂, A-7; 1♂, I-6; 1♂, SJ-2; 1♂ (IZBE), KK-1; 1♂ 2♀♀, MM-12; 1♂, K-6. Total: 13♂♂ 2♀♀.

###### Distribution in Georgia.

Samegrelo-Zemo Svanethi, Adjara, Imereti, Samtskhe-Javakheti, Kvemo Kartli, Mtskhetha-Mthianethi, Kakheti.

###### General distribution.

Palaearctic.

##### 123. Cordyla crassicornis

Taxon classificationAnimaliaDipteraMycetophilidae

Meigen, 1818

98859894-CF0A-5CCA-99AE-B7336E46AF5A

###### Material.

1♂, I-1; 1♂, I-2; 1♂, I-6; 1♂, I-11; 1♂, MM-14; 1♂, K-4; 1♂, K-6. Total: 7♂♂.

###### Distribution in Georgia.

Imereti, Mtskhetha-Mthianethi, Kakheti.

###### General distribution.

Palaearctic.

##### 124. Cordyla fasciata

Taxon classificationAnimaliaDipteraMycetophilidae

Meigen, 1830

612AFC42-AB8E-51B4-864A-C171A967DEEE

###### Material.

1♂, MM-7. Total: 1♂.

###### Distribution in Georgia.

Mtskhetha-Mthianethi.

###### General distribution.

Palaearctic.

##### 125. Cordyla fusca

Taxon classificationAnimaliaDipteraMycetophilidae

Meigen, 1804

7A02F321-D0E3-5938-B69E-1313C875004C

###### Material.

2♂♂, SJ-2 (1♂ ZFMK, 1♂ IZBE). Total: 2♂♂.

###### Distribution in Georgia.

Samtskhe-Javakheti.

###### General distribution.

Palaearctic.

##### 126. Cordyla fissa

Taxon classificationAnimaliaDipteraMycetophilidae

Edwards, 1925

26D01AB0-5E09-5E14-AAC1-78A6ED29B968

Fig. 12D

###### Material.

1♂, I-2; 1♂, I-3 (18.v–1.vi.2013); 1♂, SJ-7; 1♂, KK-1. Total: 4♂♂.

###### Distribution in Georgia.

Imereti, Samtskhe-Javakheti, Kvemo Kartli.

###### General distribution.

Palaearctic.

##### 127. Cordyla insons

Taxon classificationAnimaliaDipteraMycetophilidae

Laštovka & Matile, 1974

501FCD44-1E4B-503A-B137-326732685DF8

###### Material.

2♂♂, SJ-2 (1♂ ZFMK, 1♂ IZBE). Total: 2♂♂.

###### Distribution in Georgia.

Samtskhe-Javakheti.

###### General distribution.

Palaearctic.

##### 128. Cordyla murina

Taxon classificationAnimaliaDipteraMycetophilidae

(Winnertz, 1863)

49F49B66-89CD-5DA2-9461-D85699A863A2

###### Material.

2♂♂, I-3 (18.v–1.vi.2013 and 5–19.x.2013); 1♂, SJ-12. Total: 3♂♂.

###### Distribution in Georgia.

Samtskhe-Javakheti.

###### General distribution.

Palaearctic.

##### 129. Cordyla nitidula

Taxon classificationAnimaliaDipteraMycetophilidae

Edwards, 1925

24192B6E-4153-5B0F-BD08-1BB35EF5D9BF

###### Material.

1♂, I-14; 1♂, K-5. Total: 2♂♂.

###### Distribution in Georgia.

Imereti, Kakheti.

###### General distribution.

Palaearctic.

##### 130. Cordyla pusilla

Taxon classificationAnimaliaDipteraMycetophilidae

Edwards, 1925

360CB74B-D21E-57CD-9E2E-02A06751DF5D

###### Material.

3♂♂, SZS-3 (IZBE); 45♂♂, SZS-4 (16♂♂ ZFMK, 16♂♂ IUTG, 13♂♂ IZBE); 2♂♂, I-6; 2♂♂, K-4; 1♂, K-6. Total: 53♂♂.

###### Distribution in Georgia.

Samegrelo-Zemo Svanethi, Imereti, Kakheti.

###### General distribution.

Palaearctic.

##### 131. Exechia bicincta

Taxon classificationAnimaliaDipteraMycetophilidae

(Staeger, 1840)

A71DB4AC-2C11-5165-AC25-44BCBE77EB9F

###### Material.

4♂♂, A-5; 3♂♂, A-7; 1♂, I-9; 1♂, I-10; 1♂, I-11; 1♂, I-12; 1♂, MM-8; 1♂, MM-14; 1♂, K-2. Total: 14♂♂.

###### Distribution in Georgia.

Adjara, Imereti, Mtskhetha-Mthianethi, Kakheti.

###### General distribution.

Holarctic.

###### Remarks.

In Transcaucasia recorded from Azerbaijan (Zaitzev 2003).

##### 132. Exechia dentata

Taxon classificationAnimaliaDipteraMycetophilidae

Lundström, 1916

626B5C75-0A96-54CC-B0CE-008D92114B84

###### Material.

1♂, A-7. Total: 1♂.

###### Distribution in Georgia.

Adjara.

###### General distribution.

Europe.

##### 133. Exechia dorsalis

Taxon classificationAnimaliaDipteraMycetophilidae

(Staeger, 1840)

19A91249-D44A-518A-9296-3948FED12795

###### Material.

1♂, SJ-7; 2♂♂, MM-12; 1♂, MM-13; 3♂♂, MM-14. Total: 7♂♂.

###### Distribution in Georgia.

Samtskhe-Javakheti, Mtskhetha-Mthianethi.

###### General distribution.

Palaearctic.

##### 134. Exechia fusca

Taxon classificationAnimaliaDipteraMycetophilidae

(Meigen, 1804)

90573DFF-C175-5755-A3A3-5885415B8DCA

###### Material.

2♂♂, I-6; 1♂, SJ-3; 2♂♂, MM-2; 1♂, MM-8; 2♂♂, MM-12. Total: 8♂♂.

###### Distribution in Georgia.

Imereti, Samtskhe-Javakheti, Mtskhetha-Mthianethi.

###### General distribution.

Holarctic.

##### 135. Exechia repanda

Taxon classificationAnimaliaDipteraMycetophilidae

Johannsen, 1912

4313126A-4BD6-5D00-BD0B-A8C03AF7ADCF

###### Material.

2♂♂, K-4. Total: 2♂♂.

###### Distribution in Georgia.

Kakheti.

###### General distribution.

Holarctic.

##### 136. Exechia repandoides

Taxon classificationAnimaliaDipteraMycetophilidae

Caspers, 1984

5D451BCF-0935-567E-8CC1-C0410060BA90

###### Material.

1♂, A-3; 1♂, I-3 (24.viii–7.ix.2013); 1♂, SJ-4; 1♂, MM-12; 1♂, K-5. Total: 5♂♂.

###### Distribution in Georgia.

Adjara, Imereti, Samtskhe-Javakheti, Mtskhetha-Mthianethi, Kakheti.

###### General distribution.

Europe

##### 137. Exechia seriata

Taxon classificationAnimaliaDipteraMycetophilidae

(Meigen, 1830)

7C6A136E-A13F-5BD5-94BE-0BDE6566B4C2

###### Material.

1♂, A-5. Total: 1♂.

###### Distribution in Georgia.

Adjara.

###### General distribution.

Palaearctic.

##### 138. Exechiopsis (Exechiopsis) dumitrescae

Taxon classificationAnimaliaDipteraMycetophilidae

(Burghele-Balacesco, 1972)

DC45407F-05C5-5889-8FB9-2080F4E812F3

###### Material.

1♂, I-12. Total: ♂.

###### Distribution in Georgia.

Imereti.

###### General distribution.

Palaearctic.

##### 139. Exechiopsis (Exechiopsis) furcata

Taxon classificationAnimaliaDipteraMycetophilidae

(Lundström, 1911)

7B45499B-1357-5C76-83C4-27E546DD0E4C

###### Material.

1♂ 1♀, MM-1. Total: 1♂ 1♀.

###### Distribution in Georgia.

Mtskhetha-Mthianethi.

###### General distribution.

Europe.

##### 140. Exechiopsis (Exechiopsis) pseudindecisa

Taxon classificationAnimaliaDipteraMycetophilidae

Laštovka & Matile, 1974

89ACE343-4B65-56D1-BC12-532866737E48

###### Material.

5♂♂, MM-2. Total: 5♂♂.

###### Distribution in Georgia.

Mtskhetha-Mthianethi.

###### General distribution.

Palaearctic.

###### Remarks.

In Transcaucasia recorded from Armenia (Joost and Plassmann 1985).

##### 141. Exechiopsis (Exechiopsis) magnicauda

Taxon classificationAnimaliaDipteraMycetophilidae

(Lundström, 1911)

4F73EF82-103B-5EF8-BC26-2B9AC667EE23

###### Material.

1♂, MM-8; 2♂♂, MM-11. Total: 3♂♂.

###### Distribution in Georgia.

Mtskhetha-Mthianethi.

###### General distribution.

Europe.

##### 142. Notolopha cristata

Taxon classificationAnimaliaDipteraMycetophilidae

(Staeger, 1840)

31C34051-D7C7-56A5-A8CE-8402001B612D

###### Material.

2♂♂, SJ-8; 1♂, SJ-9. Total: 3♂♂.

###### Distribution in Georgia.

Samtskhe-Javakheti.

###### General distribution.

Holarctic.

##### 143. Pseudexechia tuomikoskii

Taxon classificationAnimaliaDipteraMycetophilidae

Kjærandsen, 2009

60FA453D-A68E-560D-9FBC-6A980A27C1BA

###### Material.

1♂, I-10. Total: 1♂.

###### Distribution in Georgia.

Imereti.

###### General distribution.

Europe.

##### 144. Rymosia affinis

Taxon classificationAnimaliaDipteraMycetophilidae

Winnertz, 1863

0C322548-4A24-5C17-9EF0-89D6D753F517

Fig. 12B

###### Material.

1♂, SJ-8. Total: 1♂.

###### Distribution in Georgia.

Samtskhe-Javakheti.

###### General distribution.

Palaearctic.

##### 145. Rymosia fasciata

Taxon classificationAnimaliaDipteraMycetophilidae

(Meigen, 1804)

51E1C0F0-6F0F-5F32-B31B-2F4BF1E40690

###### Material.

1♂, I-9. Total: 1♂.

###### Distribution in Georgia.

Imereti.

###### General distribution.

Europe.

##### 146. Stigmatomeria crassicornis

Taxon classificationAnimaliaDipteraMycetophilidae

(Stannius, 1831)

D1CEF64C-BE60-5867-8248-BE4BD5732CB0

###### Material.

1♂, A-5; 1♂, SK-1; 1♂, SJ-3; 5♂♂, SJ-8; 1♂, MM-2; 8♂♂, MM-8; 5♂♂, MM-11; 4♂♂, MM-12. Total: 26♂♂.

###### Distribution in Georgia.

Adjara, Shida Kartli, Samtskhe-Javakheti, Mtskhetha-Mthianethi.

###### General distribution.

Holarctic.

##### 147. Synplasta venosa

Taxon classificationAnimaliaDipteraMycetophilidae

(Dziedzicki, 1910)

5EC4A53E-FB93-58BD-A179-BA75D53378FE

###### Material.

1♂, A-1. Total: 1♂.

###### Distribution in Georgia.

Adjara.

###### General distribution.

Europe.

##### 148. Tarnania fenestralis

Taxon classificationAnimaliaDipteraMycetophilidae

(Meigen, 1838)

AC653FDF-F281-5499-BD1A-B2FF40849186

###### Material.

1♂, SJ-7. Total: 1♂.

###### Distribution in Georgia.

Samtskhe-Javakheti.

###### General distribution.

Palaearctic.

#### Tribe Mycetophilini

##### 149. Dynatosoma cochleare

Taxon classificationAnimaliaDipteraMycetophilidae

Strobl, 1895

C879A0BE-D234-5323-8E6F-AEBAD1FC4B71

###### Material.

1♂, SJ-8. Total: 1♂.

###### Distribution in Georgia.

Samtskhe-Javakheti.

###### General distribution.

Palaearctic.

##### 150. Dynatosoma fuscicorne

Taxon classificationAnimaliaDipteraMycetophilidae

(Meigen, 1818)

E83C84B8-F512-52E6-B493-AEFC2C45691B

###### Material.

1♂, SZS-3 (ZFMK). Total: 1♂.

###### Distribution in Georgia.

Samegrelo-Zemo Svanethi.

###### General distribution.

Holarctic.

##### 151. Dynatosoma majus

Taxon classificationAnimaliaDipteraMycetophilidae

Landrock, 1912

F0BAFAF3-7111-5048-87B9-B1354BECCF6F

###### Material.

1♀, SJ-8; 4♂♂ 1♀, MM-8; 1♂, K-6. Total: 5♂♂ 2♀♀.

###### Distribution in Georgia.

Samtskhe-Javakheti, Mtskhetha-Mthianethi, Kakheti.

###### General distribution.

Palaearctic.

##### 152. Dynatosoma nigromaculatum

Taxon classificationAnimaliaDipteraMycetophilidae

Lundström, 1913

50C45C2B-DE35-5464-A27A-83AD8C6B2C9D

###### Material.

1♀, I-6. Total: 1♀.

###### Distribution in Georgia.

Imereti.

###### General distribution.

Palaearctic.

##### 153. Dynatosoma reciprocum

Taxon classificationAnimaliaDipteraMycetophilidae

(Walker, 1848)

447934FC-6655-505B-AA40-4CDE3F7E998D

Fig. 12K

###### Material.

1♂, SJ-8. Total: 1♂.

###### Distribution in Georgia.

Samtskhe-Javakheti.

###### General distribution.

Palaearctic.

##### 154. Dynatosoma rufescens

Taxon classificationAnimaliaDipteraMycetophilidae

(Zetterstedt, 1838)

282B4262-4F7D-5F0E-91BE-A193474E650B

###### Material.

1♂, K-6. Total: 1♂.

###### Distribution in Georgia.

Kakheti.

###### General distribution.

Europe.

##### 155. Epicypta limnophila

Taxon classificationAnimaliaDipteraMycetophilidae

Chandler, 1981

ABC06099-8A31-5F57-ADDF-DF21CA285040

###### Material.

1♂, K-5. Total: 1♂.

###### Distribution in Georgia.

Kakheti.

###### General distribution.

Europe.

##### 156. Epicypta scatophora

Taxon classificationAnimaliaDipteraMycetophilidae

(Perris, 1849)

0848D148-1392-52A1-8E33-F6613DEE3D5C

Fig. 12G

###### Material.

1♂, K-4; 7♂♂ 4♀♀, K-5. Total: 8♂♂ 4♀♀.

###### Distribution in Georgia.

Kakheti.

###### General distribution.

Palaearctic.

##### 157. Epicypta torquata

Taxon classificationAnimaliaDipteraMycetophilidae

Matile, 1977

68336123-D4DB-583D-B420-E55E30FBD607

###### Material.

1♂, A-1; 1♂ 1♀, I-6; 1♂, MM-14; 1♂; K-2. Total: 4♂♂ 1♀.

###### Distribution in Georgia.

Adjara, Imereti, Mtskhetha-Mthianethi, Kakheti.

###### General distribution.

Western Palaearctic.

##### 158. Macrobrachius kowarzii

Taxon classificationAnimaliaDipteraMycetophilidae

Dziedzicki, 1889

0892226D-9E66-5B5D-8652-1733EBC06E80

###### Material.

1♂, I-6; 1♂, K-4. Total: 2♂♂.

###### Distribution in Georgia.

Imereti, Kakheti.

###### General distribution.

Europe.

##### 159. Mycetophila adumbrata

Taxon classificationAnimaliaDipteraMycetophilidae

Mik, 1884

28F22DB6-5FAA-5B9F-9A8A-485262233C45

###### Material.

1♂, SJ-8; 1♂, MM-13. Total: 2♂♂.

###### Distribution in Georgia.

Samtskhe-Javakheti, Mtskhetha-Mthianethi.

###### General distribution.

Europe.

##### 160. Mycetophila alea

Taxon classificationAnimaliaDipteraMycetophilidae

Laffoon, 1965

ACD09D52-0029-5E65-AFD0-F735B50AACAB

###### Material.

1♂, A-3; 35♂♂, I-6; 1♂, I-9; 1♂, I-14; 1♂, SK-1; 1♂, SJ-1 (ZFMK); 3♂♂, SJ-4; 2♂♂, SJ-9; 2♂♂, MM-8; 2♂♂, MM-11; 1♂, KK-1. Total: 50♂♂.

###### Distribution in Georgia.

Adjara, Imereti, Shida Kartli, Samtskhe-Javakheti, Mtskhetha-Mthianethi, Kvemo Kartli.

###### General distribution.

Holarctic.

##### 161. Mycetophila bialorussica

Taxon classificationAnimaliaDipteraMycetophilidae

Dziedzicki, 1884

389A7EDC-456C-529F-86A2-4800B827867D

###### Material.

1♂, SZS-4 (ZFMK); 1♂, SJ-4; 1♂, SJ-8; 1♂, SJ-9. Total: 4♂♂.

###### Distribution in Georgia.

Samegrelo-Zemo Svanethi, Samtskhe-Javakheti.

###### General distribution.

Palaearctic.

##### 162. Mycetophila blanda

Taxon classificationAnimaliaDipteraMycetophilidae

Winnertz, 1863

93473FF4-C382-5CB4-858B-3748C21D5A00

###### Material.

2♂♂, I-6; 1♂, SJ-9. Total: 3♂♂.

###### Distribution in Georgia.

Imereti, Samtskhe-Javakheti.

###### General distribution.

Palaearctic.

##### 163. Mycetophila brevitarsata

Taxon classificationAnimaliaDipteraMycetophilidae

(Laštovka, 1963)

AF14881B-2BE0-546C-98F2-1BA77DDEADE9

###### Material.

1♂, SZS-4 (ZFMK). Total: 1♂.

###### Distribution in Georgia.

Samegrelo-Zemo Svanethi.

###### General distribution.

Palaearctic.

###### Remarks.

Listed to occur in Transcaucasia without further details (Zaitzev 2003).

##### 164. Mycetophila distigma

Taxon classificationAnimaliaDipteraMycetophilidae

Meigen, 1830

FDF71E32-7F08-5639-A1A8-7BF09E196F09

###### Material.

1♂, SJ-8; 1♂, K-4; Total: 2♂♂.

###### Distribution in Georgia.

Samtskhe-Javakheti, Kakheti.

###### General distribution.

Europe.

##### 165. Mycetophila edwardsi

Taxon classificationAnimaliaDipteraMycetophilidae

Lundström, 1913

CCF0FB87-9255-5834-AE2A-2666E2758C0B

###### Material.

1♂, SJ-7; 3♂♂, SJ-8; 1♂, MM-8. Total: 5♂♂.

###### Distribution in Georgia.

Samtskhe-Javakheti, Mtskhetha-Mthianethi.

###### General distribution.

Europe.

##### 166. Mycetophila exstincta

Taxon classificationAnimaliaDipteraMycetophilidae

Loew, 1869

CF7B65A6-9F99-5547-9898-71FCD1047EB9

###### Material.

2♂♂, SZS-3 (1♂ ZFMK, 1♂ IZBE); 1♂, I-14; 2♂♂, K-4. Total: 5♂♂.

###### Distribution in Georgia.

Samegrelo-Zemo Svanethi, Imereti, Kakheti.

###### General distribution.

Holarctic.

##### 167. Mycetophila formosa

Taxon classificationAnimaliaDipteraMycetophilidae

Lundström, 1911

168A1BE4-7185-55A8-A4F1-31C956DB970D

###### Material.

1♂, SJ-3; 1♂, SJ-8. Total: 2♂♂.

###### Distribution in Georgia.

Samtskhe-Javakheti.

###### General distribution.

Palaearctic.

##### 168. Mycetophila fungorum

Taxon classificationAnimaliaDipteraMycetophilidae

(De Geer, 1776)

489DC62F-B1AC-5F09-985F-4C8F8C88A7AA

###### Material.

6♂♂ 3♀♀, SZS-3 (IZBE); 19♂♂ 10♀♀, SZS-4 (9♂♂ 5♂♀ZFMK, 8♂♂ 5♀♀ IUTG, 2♂♂ IZBE); 1♂, I-3 (29.vi–13.vii.2013); 5♂♂ 2♀♀, I-6; 1♀, I-9; 1♂, I-10; 1♀, SK-1; 2♂♂, SJ-1 (IZBE); 2♀♀, SJ-3; 1♂, SJ-4; 2♂♂ 2♀♀, SJ-7; 4♂♂ 4♀♀, SJ-8; 2♂♂ 4♀♀, SJ-9; 1♂, SJ-10; 2♂♂, MM-3; 1♀, MM-6; 2♂♂ 7♀♀, MM-7; 1♂ 1♀, MM-8; 2♂♂ 2♀♀, MM-11; 1♀, MM-12; 1♂, MM-14; 1♂ 2♀♀, KK-1; 1♂, K-4. Total: 54♂♂ 43♀♀.

###### Distribution in Georgia.

Samegrelo-Zemo Svanethi, Imereti, Shida Kartli, Samtskhe-Javakheti, Mtskhetha-Mthianethi, Kvemo Kartli, Kakheti.

###### General distribution.

Holarctic (extending to the Oriental region).

##### 169. Mycetophila confluens

Taxon classificationAnimaliaDipteraMycetophilidae

Dziedzicki, 1884

DC328F65-6563-5066-B5EA-95BFF7C03AFD

###### Material.

4♂♂, SJ-8; 1♂, MM-2. Total: 5♂♂.

###### Distribution in Georgia.

Samtskhe-Javakheti, Mtskhetha-Mthianethi.

###### General distribution.

Holarctic.

##### 170. Mycetophila curviseta

Taxon classificationAnimaliaDipteraMycetophilidae

Lundström, 1911

6FB7A634-74E7-508E-B98E-40BDBF81CEF4

###### Material.

4♂♂, SZS-4 (2♂♂ ZFMK, 1♂ IUTG, 1♂ IZBE); 1♂, I-3 (1–15.vi.2013); 3♂♂, I-6; 2♂♂, I-9; 1♂, I-12; 2♂♂, SK-1; 6♂♂, SJ-8; 5♂♂, SJ-9; 1♂, KK-1; 3♂♂, MM-8; 1♂, MM-13; 7♂♂, MM-14; 3♂♂, K-4; 1♂, K-5. Total: 40♂♂.

###### Distribution in Georgia.

Samegrelo-Zemo Svanethi, Imereti, Shida Kartli, Samtskhe-Javakheti, Kvemo Kartli, Mtskhetha-Mthianethi, Kakheti.

###### General distribution.

Palaearctic.

##### 171. Mycetophila deflexa

Taxon classificationAnimaliaDipteraMycetophilidae

Chandler, 2001

8B540984-5893-5E81-A541-6CCF5D7D40D0

###### Material.

2♂♂, SZS-3 (1♂ ZFMK, 1♂ IZBE); 1♂, SZS-4 (IUTG). Total: 3♂♂.

###### Distribution in Georgia.

Samegrelo-Zemo Svanethi.

###### General distribution.

Europe.

##### 172. Mycetophila dentata

Taxon classificationAnimaliaDipteraMycetophilidae

Lundström, 1915

65C6AC2E-544B-53A4-BA47-FAD0BDAB78FE

###### Material.

1♂, K-4. Total: 1♂.

###### Distribution in Georgia.

Kakheti.

###### General distribution.

Holarctic.

##### 173. Mycetophila gentilicia

Taxon classificationAnimaliaDipteraMycetophilidae

Zaitzev, 1999

00E196B0-0114-5213-8AE1-F3B4BCE591C2

###### Material.

1♂, SJ-8. Total: 1♂.

###### Distribution in Georgia.

Samtskhe-Javakheti.

###### General distribution.

Palaearctic.

##### 174. Mycetophila gibbula

Taxon classificationAnimaliaDipteraMycetophilidae

Edwards, 1925

4B87C45E-E36F-5E12-9C8F-C919B00B0AA3

###### Material.

1♂, SJ-3. Total: 1♂.

###### Distribution in Georgia.

Samtskhe-Javakheti.

###### General distribution.

Palaearctic.

##### 175. Mycetophila hetschkoi

Taxon classificationAnimaliaDipteraMycetophilidae

Landrock 1918

2BA23DE1-BF91-52C2-BB7A-1CEAAE498D47

###### Material.

1♂, SZS-4 (ZFMK); 1♂, MM-11; 3♂♂, MM-14. Total: 5♂♂.

###### Distribution in Georgia.

Samegrelo-Zemo Svanethi, Mtskhetha-Mthianethi.

###### General distribution.

Palaearctic.

##### 176. Mycetophila hyrcania

Taxon classificationAnimaliaDipteraMycetophilidae

Laštovka & Matile, 1969

1C8429B7-C99B-5C74-90A8-7121923B5E3E

###### Material.

1♂, SZS-3 (ZFMK); 1♂, SJ-6; 1♂, KK-1; 3♂♂, K-4. Total: 6♂♂.

###### Distribution in Georgia.

Samegrelo-Zemo Svanethi, Samtskhe-Javakheti, Kvemo Kartli, Kakheti.

###### General distribution.

Western Palaearctic.

##### 177. Mycetophila ichneumonea

Taxon classificationAnimaliaDipteraMycetophilidae

Say, 1823

85DE223E-7BBD-5DF0-8DFF-35673AAE6E5F

###### Georgian source.

Jürgenstein et al. 2015: 30.

###### Material.

6♂♂, SZS-4 (3♂♂ ZFMK, 2♂♂ IUTG, 1♂ IZBE); 1♂, SK-1; 1♂, SJ-2 (IZBE); 1♂, SJ-5; 3♂♂, SJ-8; 1♂, SJ-9. Total: 13♂♂.

###### Distribution in Georgia.

Samegrelo-Zemo Svanethi, Shida Kartli, Samtskhe-Javakheti.

###### General distribution.

Holarctic. Listed to occur in Transcaucasia without further details (Zaitzev 2003).

##### 178. Mycetophila idonea

Taxon classificationAnimaliaDipteraMycetophilidae

Laštovka, 1972

76A14A57-5F05-540F-A8CF-4BFD42F995C0

###### Georgian source.

Jürgenstein et al. 2015: 31–32.

###### Material.

5♂♂, SZS-3 (2♂♂ ZFMK, 2♂♂ IUTG, 1♂ IZBE); 2♂♂, A-1; 1♂, A-3; 1♂, A-7; 10♂♂, I-6; 1♂, I-10; 1♂, SK-1; 1♂, SJ-1 (IZBE); 1♂, KK-1; 1♂, MM-7; 1♂, MM-8; 4♂♂, MM-11; 1♂, MM-14; 1♂, K-4; 1♂, K-5; 1♂, K-6. Total: 33♂♂.

###### Distribution in Georgia.

Samegrelo-Zemo Svanethi, Adjara, Imereti, Shida Kartli, Samtskhe-Javakheti, Kvemo Kartli, Mtskhetha-Mthianethi, Kakheti.

###### General distribution.

Europe.

##### 179. Mycetophila lamellata

Taxon classificationAnimaliaDipteraMycetophilidae

Lundström, 1911

8212DE16-906B-59CE-B68E-382E3D382ECF

###### Material.

4♂♂, SJ-7; 1♂, SJ-8; 2♂♂, MM-14. Total: 7♂♂.

###### Distribution in Georgia.

Samtskhe-Javakheti, Mtskhetha-Mthianethi.

###### General distribution.

Europe.

##### 180. Mycetophila lastovkai

Taxon classificationAnimaliaDipteraMycetophilidae

Caspers, 1984

B2FC8EFB-E1FC-5EA3-A6D0-DD106AA16E5A

###### Material.

1♂, SZS-4 (ZFMK). Total: 1♂.

###### Distribution in Georgia.

Samegrelo-Zemo Svanethi.

###### General distribution.

Europe.

##### 181. Mycetophila luctuosa

Taxon classificationAnimaliaDipteraMycetophilidae

Meigen, 1830

CC697F16-89E7-5A5B-88CF-318D45B9B017

###### Material.

1♂, SZS-4 (ZFMK); 1♂, I-10; 1♂, MM-4. Total: 3♂♂.

###### Distribution in Georgia.

Samegrelo-Zemo Svanethi, Imereti, Mtskhetha-Mthianethi.

###### General distribution.

Holarctic.

##### 182. Mycetophila lunata

Taxon classificationAnimaliaDipteraMycetophilidae

Meigen, 1804

4EF8C3FA-B79B-5164-B32F-E659BB7E8A1D

###### Material.

1♂, A-1; 1♂, MM-12. Total: 2♂♂.

###### Distribution in Georgia.

Adjara, Mtskhetha-Mthianethi.

###### General distribution.

Palaearctic.

##### 183. Mycetophila magnicauda

Taxon classificationAnimaliaDipteraMycetophilidae

Strobl, 1895

2712016D-F608-5198-BFA1-E8C67314F93F

Fig. 12F

###### Material.

1♂, SJ-4; 4♂♂, SJ-8; 1♂, SJ-9. Total: 6♂♂.

###### Distribution in Georgia.

Samtskhe-Javakheti.

###### General distribution.

Palaearctic.

##### 184. Mycetophila marginata

Taxon classificationAnimaliaDipteraMycetophilidae

Winnertz, 1863

069A0A42-0C58-5516-B00F-08F7B2D5DF38

###### Material.

1♂, I-6; 1♂, I-10; 4♂♂, SJ-7; 5♂♂, SJ-8; 2♂♂, SJ-9; 2♂♂, MM-2. Total: 15♂♂.

###### Distribution in Georgia.

Imereti, Samtskhe-Javakheti, Mtskhetha-Mthianethi.

###### General distribution.

Europe.

##### 185. Mycetophila morosa

Taxon classificationAnimaliaDipteraMycetophilidae

Winnertz, 1863

E45125F4-298B-596A-936B-F1B731C7C893

###### Material.

1♂, MM-14. Total: 1♂.

###### Distribution in Georgia.

Samegrelo-Zemo Svanethi.

###### General distribution.

Holarctic.

##### 186. Mycetophila nigrofusca

Taxon classificationAnimaliaDipteraMycetophilidae

Dziedzicki, 1884

D7DAFBD5-359D-5476-A192-0461D292B49A

###### Material.

1♂, I-5; 1♂, MM-14. Total: 2♂♂.

###### Distribution in Georgia.

Imereti, Mtskhetha-Mthianethi.

###### General distribution.

Palaearctic.

##### 187. Mycetophila ocellus

Taxon classificationAnimaliaDipteraMycetophilidae

Walker, 1848

F324D5A3-A78F-53F0-83D5-7D7E41A15B75

###### Material.

1♂, I-10; 3♂♂, SJ-8; 2♂♂, MM-12. Total: 6♂♂.

###### Distribution in Georgia.

Imereti, Samtskhe-Javakheti, Mtskhetha-Mthianethi.

###### General distribution.

Holarctic.

##### 188. Mycetophila occultans

Taxon classificationAnimaliaDipteraMycetophilidae

Lundström, 1913

4B62F2C8-AEB8-593D-B92F-D59E9CB0194F

###### Material.

6♂♂, SZS-3 (2♂♂ ZFMK, 2♂♂ IUTG, 2♂♂ IZBE);1♂, I-6; 1♂, SJ-8; 1♂, SJ-9; 57♂♂, MM-13; 1♂, MM-14. Total: 67♂♂.

###### Distribution in Georgia.

Samegrelo-Zemo Svanethi, Imereti, Samtskhe-Javakheti, Mtskhetha-Mthianethi.

###### General distribution.

Europe.

##### 189. Mycetophila ornata

Taxon classificationAnimaliaDipteraMycetophilidae

Stephens, 1829

507338BC-5E2D-56BA-B18B-E401D5249F87

###### Material.

2♂♂, SJ-8; 1♂, SJ-9; 2♂♂, MM-12. Total: 5♂♂.

###### Distribution in Georgia.

Samtskhe-Javakheti, Mtskhetha-Mthianethi.

###### General distribution.

Palaearctic.

###### Remarks.

In Transcaucasia recorded from Azerbaijan (Zaitzev 2003).

##### 190. Mycetophila pictula

Taxon classificationAnimaliaDipteraMycetophilidae

Meigen, 1830

7FDFAE45-E491-5B7D-8D7C-67D8A45A647B

###### Material.

2♂♂, SJ-9. Total: 2♂♂.

###### Distribution in Georgia.

Samtskhe-Javakheti.

###### General distribution.

Holarctic.

##### 191. Mycetophila pumila

Taxon classificationAnimaliaDipteraMycetophilidae

Winnertz, 1863

6C5B32A1-D503-51F2-B884-37E0A458D6F6

###### Material.

1♂, SZS-4 (ZFMK); 2♂♂, A-1; 2♂♂, A-3; 3♂♂, I-6; 1♂, I-9; 1♂, I-10; 1♂, MM-11; 4♂♂, MM-14; 1♂, K-2. Total: 16♂♂.

###### Distribution in Georgia.

Samegrelo-Zemo Svanethi, Adjara, Imereti, Mtskhetha-Mthianethi, Kakheti.

###### General distribution.

Palaearctic.

##### 192. Mycetophila pseudoforcipata

Taxon classificationAnimaliaDipteraMycetophilidae

Zaitzev, 1998

453F08B4-5EA0-5B4A-878E-C1D5369D5A82

###### Material.

1♂, SJ-8; 1♂, SJ-9. Total: 2♂♂.

###### Distribution in Georgia.

Samtskhe-Javakheti.

###### General distribution.

Palaearctic.

##### 193. Mycetophila ruficollis

Taxon classificationAnimaliaDipteraMycetophilidae

Meigen, 1818

B415D45E-7D3E-5CA3-9DF2-023E6C2BB15A

###### Georgian source.

Jürgenstein et al. 2015: 33.

###### Material.

1♂, K-6. Total: 1♂.

###### Distribution in Georgia.

Kakheti.

###### General distribution.

Palaearctic.

##### 194. Mycetophila scotica

Taxon classificationAnimaliaDipteraMycetophilidae

Edwards, 1941

A15A1C1E-33AD-5875-B014-35551E58BDF0

###### Material.

1♂, I-12. Total: 1♂.

###### Distribution in Georgia.

Imereti.

###### General distribution.

Holarctic.

##### 195. Mycetophila sigillata

Taxon classificationAnimaliaDipteraMycetophilidae

Dziedzicki, 1884

84523617-099F-5544-9D00-98FBD5D4EC29

###### Material.

3♂♂, I-6; 2♂♂, SJ-4. Total: 5♂♂.

###### Distribution in Georgia.

Imereti, Samtskhe-Javakheti.

###### General distribution.

Holarctic.

###### Remarks.

Listed to occur in Transcaucasia without further details (Zaitzev 2003).

##### 196. Mycetophila sigmoides

Taxon classificationAnimaliaDipteraMycetophilidae

Loew, 1869

A4ABFEFD-FFE8-5B71-9065-8BDB1DD9DAF2

###### Material.

1♂, I-6. Total: 1♂.

###### Distribution in Georgia.

Imereti.

###### General distribution.

Holarctic.

##### 197. Mycetophila signata

Taxon classificationAnimaliaDipteraMycetophilidae

Meigen, 1830

DB1CA734-9D81-5F92-8505-596C8EA2B61C

###### Material.

10♂♂, I-6; 1♂, I-14; 1♂, SJ-4. Total: 12♂♂.

###### Distribution in Georgia.

Imereti, Samtskhe-Javakheti.

###### General distribution.

Palaearctic.

##### 198. Mycetophila signatoides

Taxon classificationAnimaliaDipteraMycetophilidae

Dziedzicki, 1884

786E2002-D0C5-5564-9975-9B362DC69448

###### Material.

1♂, SZS-4 (ZFMK); 2♂♂, I-6. Total: 3♂♂.

###### Distribution in Georgia.

Samegrelo-Zemo Svanethi, Imereti.

###### General distribution.

Western Palaearctic (see also comment in Kjaerandsen et al. 2007).

##### 199. Mycetophila

Taxon classificationAnimaliaDipteraMycetophilidae

sordida van der Wulp, 1874

5D4E6C13-B7A2-59EF-B098-63492EDC3F8C

###### Material.

1♂, SZS-3 (ZFMK); 3♂♂, SZS-4 (1♂ ZFMK, 1♂ IUTG, 1♂ IZBE); 2♂♂, I-6; 1♂, SJ-4; 4♂♂, SJ-8; 1♂, SJ-9; 6♂♂, KK-1; 1♂, MM-11. Total: 19♂♂.

###### Distribution in Georgia.

Samegrelo-Zemo Svanethi, Imereti, Samtskhe-Javakheti, Kvemo Kartli, Mtskhetha-Mthianethi.

###### General distribution.

Holarctic.

##### 200. Mycetophila strigatoides

Taxon classificationAnimaliaDipteraMycetophilidae

Landrock, 1927

4CA5DAE2-8C28-5A68-924A-D11C6FC9C6DF

###### Material.

4♂♂, SZS-3 (2♂♂ ZFMK, 2♂♂ IZBE); 42♂♂, SZS-4 (13♂♂ ZFMK, 16♂♂ IUTG, 13♂♂ IZBE); 1♂, I-10; 1♂, SJ-1 (IZBE); 1♂, SJ-2 (ZFMK). Total: 49♂♂.

###### Distribution in Georgia.

Samegrelo-Zemo Svanethi, Imereti, Samtskhe-Javakheti.

###### General distribution.

Palaearctic.

##### 201. Mycetophila stylata

Taxon classificationAnimaliaDipteraMycetophilidae

(Dziedzicki, 1884)

BD23232A-7516-5A32-883F-C4954A2F2405

###### Material.

9♂♂, SJ-8; 4♂♂, SJ-9. Total: 13♂♂.

###### Distribution in Georgia.

Samtskhe-Javakheti.

###### General distribution.

Palaearctic.

##### 202. Mycetophila sublunata

Taxon classificationAnimaliaDipteraMycetophilidae

Zaitzev, 1998

69C3EFEE-8D2C-5945-A872-F526D23FF616

###### Material.

1♂, SJ-9. Total: 1♂.

###### Distribution in Georgia.

Samtskhe-Javakheti.

###### General distribution.

Europe.

##### 203. Mycetophila subsigillata

Taxon classificationAnimaliaDipteraMycetophilidae

Zaitzev, 1999

68D3862B-E35D-5A93-AAEC-DD76050A7DE0

###### Material.

1♂, SJ-8. Total: 1♂.

###### Distribution in Georgia.

Samtskhe-Javakheti.

###### General distribution.

Palaearctic.

##### 204. Mycetophila sumavica

Taxon classificationAnimaliaDipteraMycetophilidae

(Laštovka, 1963)

29B8AD76-6C64-5F7D-A53C-A34D4823B281

###### Material.

1♂, I-10. Total: 1♂.

###### Distribution in Georgia.

Imereti.

###### General distribution.

Europe.

##### 205. Mycetophila trinotata

Taxon classificationAnimaliaDipteraMycetophilidae

Staeger, 1840

2AB9613F-06D8-52B9-8DBD-8430B6414A35

###### Material.

1♂, SZS-3 (ZFMK); 5♂♂, MM-13; 12♂♂, MM-14; 1♂, K-2. Total: 19♂♂.

###### Distribution in Georgia.

Samegrelo-Zemo Svanethi, Mtskhetha-Mthianethi, Kakheti.

###### General distribution.

Holarctic.

##### 206. Mycetophila uliginosa

Taxon classificationAnimaliaDipteraMycetophilidae

Chandler, 1988

FAB2160F-F6A7-5289-8B33-173651B55C30

###### Material.

1♂, SZS-4 (ZFMK). Total: 1♂.

###### Distribution in Georgia.

Samegrelo-Zemo Svanethi.

###### General distribution.

Europe.

##### 207. Mycetophila unicolor

Taxon classificationAnimaliaDipteraMycetophilidae

Stannius, 1831

2608ACC1-ABCA-5320-865F-689BB5CA3207

###### Material.

3♂♂, A-1; 2♂♂, A-7; 1♂, SJ-2 (ZFMK). Total: 6♂♂.

###### Distribution in Georgia.

Adjara, Samtskhe-Javakheti.

###### General distribution.

Western Palaearctic.

##### 208. Phronia basalis

Taxon classificationAnimaliaDipteraMycetophilidae

Winnertz, 1863

1A872E82-3849-5260-93D8-1B73E8288457

###### Material.

1♂, MM-10. Total: 1♂.

###### Distribution in Georgia.

Mtskhetha-Mthianethi.

###### General distribution.

Western Palaearctic.

##### 209. Phronia biarcuata

Taxon classificationAnimaliaDipteraMycetophilidae

(Becker, 1908)

F77F38C2-E1BC-5C21-80E6-2D90D2622030

###### Material.

1♂, SJ-8; 1♂, SJ-9; 1♂, MM-12. Total: 3♂♂.

###### Distribution in Georgia.

Samtskhe-Javakheti, Mtskhetha-Mthianethi.

###### General distribution.

Holarctic.

###### Remarks.

In Transcaucasia recorded from Armenia (Joost and Plassmann 1985).

##### 210. Phronia conformis

Taxon classificationAnimaliaDipteraMycetophilidae

(Walker, 1856)

72FD0674-EA83-5E11-B8C2-5811E31D493C

###### Material.

2♂♂, I-6. Total: 2♂♂.

###### Distribution in Georgia.

Imereti.

###### General distribution.

Holarctic.

##### 211. Phronia electa

Taxon classificationAnimaliaDipteraMycetophilidae

Dziedzicki, 1889

0DAC051C-2CF8-5F92-9A7F-18F84423308A

###### Material.

1♂, SJ-8. Total: 1♂.

###### Distribution in Georgia.

Samtskhe-Javakheti.

###### General distribution.

Palaearctic.

##### 212. Phronia exigua

Taxon classificationAnimaliaDipteraMycetophilidae

(Zetterstedt, 1852)

FF0C737F-E055-5213-BC20-1E80FD73C7DC

###### Material.

2♂♂, MM-2; 1♂, MM-8. Total: 3♂♂.

###### Distribution in Georgia.

Mtskhetha-Mthianethi.

###### General distribution.

Holarctic.

###### Remarks.

Listed to occur in Transcaucasia without further details (Zaitzev 2003).

##### 213. Phronia humeralis

Taxon classificationAnimaliaDipteraMycetophilidae

Winnertz, 1863

38D49FE8-D199-5D32-A78B-97CC0F7989C7

###### Material.

1♂, A-7; 1♂, SJ-4; 2♂♂, SJ-8; 2♂♂, SJ-9. Total: 6♂♂.

###### Distribution in Georgia.

Adjara, Samtskhe-Javakheti.

###### General distribution.

Palaearctic.

##### 214. Phronia forcipata

Taxon classificationAnimaliaDipteraMycetophilidae

Winnertz, 1863

BAD7C9FD-3106-5F5D-ACF5-959E6306EFD2

###### Material.

1♂, SZS-4 (ZFMK); 8♂♂, I-6. Total: 9♂♂.

###### Distribution in Georgia.

Samegrelo-Zemo Svanethi, Imereti.

###### General distribution.

Palaearctic.

###### Remarks.

Listed to occur in Transcaucasia without further details (Zaitzev 2003).

##### 215. Phronia nitidiventris

Taxon classificationAnimaliaDipteraMycetophilidae

(van der Wulp, 1859)

C2464E28-4DA3-5FD0-A233-962B20CBA232

###### Material.

2♂♂, I-6; 1♂, I-9; 1♂, I-10. Total: 4♂♂.

###### Distribution in Georgia.

Imereti.

###### General distribution.

Palaearctic.

###### Remarks.

Listed to occur in Transcaucasia without further details (Zaitzev 2003).

##### 216. Phronia notata

Taxon classificationAnimaliaDipteraMycetophilidae

Dziedzicki, 1889

D9887017-89FA-5254-8022-6B772244F284

###### Material.

1♂, SJ-8. Total: 1♂.

###### Distribution in Georgia.

Samtskhe-Javakheti.

###### General distribution.

Palaearctic.

##### 217. Phronia obtusa

Taxon classificationAnimaliaDipteraMycetophilidae

Winnertz, 1863

53D73012-7EE7-598C-AD68-791855EA8D62

###### Material.

1♂, I-6. Total: 1♂.

###### Distribution in Georgia.

Imereti.

###### General distribution.

Holarctic.

##### 218. Phronia petulans

Taxon classificationAnimaliaDipteraMycetophilidae

Dziedzicki, 1889

334D7AFA-1FFA-5334-94C0-96AD497F9AC6

###### Material.

6♂♂, MM-12. Total: 6♂♂.

###### Distribution in Georgia.

Mtskhetha-Mthianethi.

###### General distribution.

Holarctic.

##### 219. Phronia signata

Taxon classificationAnimaliaDipteraMycetophilidae

Winnertz, 1863

2BBFDCF1-AEBB-5E4B-B4F4-909F69F5933A

###### Material.

9♂♂, I-6; 1♂, I-9; 4♂♂, I-10; 1♂, SJ-9; 8♂♂, MM-8; 1♂, MM-9. Total: 24♂♂.

###### Distribution in Georgia.

Imereti, Samtskhe-Javakheti, Mtskhetha-Mthianethi.

###### General distribution.

Palaearctic.

###### Remarks.

Listed to occur in Transcaucasia without further details (Zaitzev 2003).

##### 220. Phronia tenuis

Taxon classificationAnimaliaDipteraMycetophilidae

Winnertz, 1863

E5C7B49E-CF57-56B8-B83B-702EEC76D8CC

Fig. 12H

###### Material.

2♂♂, SZS-4 (1♂ ZFMK, 1♂ IZBE); 1♂, I-10; 1♂, MM-12. Total: 4♂♂.

###### Distribution in Georgia.

Samegrelo-Zemo Svanethi, Imereti, Mtskhetha-Mthianethi.

###### General distribution.

Holarctic, extending to the Oriental region.

##### 221. Phronia triangularis

Taxon classificationAnimaliaDipteraMycetophilidae

Winnertz, 1863

5D13AECB-798F-5821-9218-0D5620610149

###### Material.

1♂, I-6; 1♂, SJ-8; 1♂, MM-2; 1♂, MM-8. Total: 4♂♂.

###### Distribution in Georgia.

Imereti, Samtskhe-Javakheti, Mtskhetha-Mthianethi.

###### General distribution.

Western Europe.

##### 222. Platurocypta testata

Taxon classificationAnimaliaDipteraMycetophilidae

(Edwards, 1925)

23A0F64A-3891-5000-92D1-5437E8E489B3

###### Material.

1♂, I-3 (13–27.vi.2013); 2♂♂, SJ-4; 1♂, K-4. Total: 4♂♂.

###### Distribution in Georgia.

Imereti, Samtskhe-Javakheti, Kakheti.

###### General distribution.

Holarctic.

##### 223. Platurocypta punctum

Taxon classificationAnimaliaDipteraMycetophilidae

(Stannius, 1831)

DD780163-2E5C-5AA7-A262-6A0979937328

###### Material.

1♂, K-2. Total: 1♂.

###### Distribution in Georgia.

Kakheti.

###### General distribution.

Holarctic.

##### 224. Sceptonia cryptocauda

Taxon classificationAnimaliaDipteraMycetophilidae

Chandler, 1991

C200A2C5-524A-5A4F-8775-F72927C9AAEB

###### Material.

18♂♂, MM-13; 18♂♂, MM-14; 8♂♂, K-2. Total: 44♂♂.

###### Distribution in Georgia.

Mtskhetha-Mthianethi; Kakheti.

###### General distribution.

Western Palaearctic.

##### 225. Sceptonia demeijerei

Taxon classificationAnimaliaDipteraMycetophilidae

Bechev, 1997

3ED2F776-BB4D-5845-90D9-EB00AA6DC7BB

###### Material.

1♂, MM-5. Total: 1♂.

###### Distribution in Georgia.

Mtskhetha-Mthianethi.

###### General distribution.

Europe.

##### 226. Sceptonia flavipuncta

Taxon classificationAnimaliaDipteraMycetophilidae

Edwards, 1925

DBDAFE0F-73A7-53A5-B9E6-741DCC2CE330

###### Material.

1♂, SZS-3 (IZBE); 6♂♂, I-6; 3♂♂, I-9; 2♂♂, I-14; 1♂, SJ-2 (ZFMK); 1♂, KK-1; 10♂♂, MM-13; 14♂♂, MM-14. Total: 38♂♂.

###### Distribution in Georgia.

Samegrelo-Zemo Svanethi, Imereti, Samtskhe-Javakheti, Kvemo Kartli, Mtskhetha-Mthianethi.

###### General distribution.

Europe.

##### 227. Sceptonia humerella

Taxon classificationAnimaliaDipteraMycetophilidae

Edwards, 1925

44976C6B-5EA2-51A0-8710-4B47BC790C7B

###### Material.

1♂, SJ-3; 1♂, SJ-4. Total: 2♂♂.

###### Distribution in Georgia.

Samtskhe-Javakheti.

###### General distribution.

Europe.

##### 228. Sceptonia membranacea

Taxon classificationAnimaliaDipteraMycetophilidae

Edwards, 1925

4BB9F4C2-60C0-5920-82E8-C70D28A2F99D

###### Material.

5♂♂, MM-13. Total: 5♂♂.

###### Distribution in Georgia.

Mtskhetha-Mthianethi.

###### General distribution.

Europe.

##### 229. Sceptonia nigra

Taxon classificationAnimaliaDipteraMycetophilidae

(Meigen, 1804)

87654F15-0479-5360-ABF6-AD9806969053

###### Material.

2♂♂, A-3; 1♂, MM-13; 14♂♂, MM-14; 7♂♂, K-2. Total: 24♂♂.

###### Distribution in Georgia.

Adjara, Mtskhetha-Mthianethi, Kakheti.

###### General distribution.

Palaearctic.

##### 230. Sceptonia tenuis

Taxon classificationAnimaliaDipteraMycetophilidae

Edwards, 1925

89B4B4D0-243E-5505-907E-2A3C5725206E

Fig. 12 I

###### Material.

1♂, SK-1; 3♂♂, SJ-2 (1♂ ZFMK, 1♂ IUTG, 1♂ IZBE); 2♂♂, MM-12. Total: 6♂♂.

###### Distribution in Georgia.

Shida Kartli, Samtskhe-Javakheti, Mtskhetha-Mthianethi.

###### General distribution.

Europe.

##### 231. Trichonta aberrans

Taxon classificationAnimaliaDipteraMycetophilidae

Lundström, 1911

34C6D066-B47D-5502-9EC5-BB1B543748A0

###### Material.

1♂, I-6. Total: 1♂.

###### Distribution in Georgia.

Imereti.

###### General distribution.

Europe.

##### 232. Trichonta atricauda

Taxon classificationAnimaliaDipteraMycetophilidae

(Zetterstedt, 1852)

DEAEFA39-2B5C-5EEA-89F4-8DAB9D2D8D61

###### Material.

1♂, I-6. Total: 1♂.

###### Distribution in Georgia.

Imereti.

###### General distribution.

Holarctic.

##### 233. Trichonta clavigera

Taxon classificationAnimaliaDipteraMycetophilidae

Lundström, 1913

D5A7B4C6-076C-5583-AC0B-12A352038A11

###### Material.

2♂♂, I-6; 1♂, KK-1; 1♂, MM-12. Total: 4♂♂.

###### Distribution in Georgia.

Imereti, Kvemo Kartli, Mtskhetha-Mthianethi.

###### General distribution.

Palaearctic.

##### 234. Trichonta falcata

Taxon classificationAnimaliaDipteraMycetophilidae

Lundström, 1911

6636485D-1F0D-5DF4-A574-291CDA56AC1B

###### Material.

2♂♂, A-7. Total: 2♂♂.

###### Distribution in Georgia.

Adjara.

###### General distribution.

Holarctic.

###### Remarks.

Listed to occur in Transcaucasia without further details (Zaitzev 2003).

##### 235. Trichonta fragilis

Taxon classificationAnimaliaDipteraMycetophilidae

Gagne, 1981

13B50CBB-AAE4-57FF-99FF-08B8A6A649DC

###### Material.

1♂, I-6. Total: 1♂.

###### Distribution in Georgia.

Imereti.

###### General distribution.

Holarctic.

##### 236. Trichonta perspicua

Taxon classificationAnimaliaDipteraMycetophilidae

van der Wulp, 1881

690238D5-2DA9-5A02-B0D0-1C2C09E81FBA

###### Material.

1♂, I-17. Total: 1♂.

###### Distribution in Georgia.

Imereti.

###### General distribution.

Holarctic.

##### 237. Trichonta subterminalis

Taxon classificationAnimaliaDipteraMycetophilidae

Zaitzev & Menzel, 1996

EDFF1E14-CE1C-5D9D-9F0E-264979A5CF00

###### Material.

4♂♂, I-6; 1♂, SK-1. Total: 5♂♂.

###### Distribution in Georgia.

Imereti, Shida Kartli.

###### General distribution.

Palaearctic.

##### 238. Trichonta trifida

Taxon classificationAnimaliaDipteraMycetophilidae

Lundström, 1909

B7F405DA-A8A0-5447-85D1-466748A9FCD4

###### Material.

1♂, I-10. Total: 1♂.

###### Distribution in Georgia.

Imereti.

###### General distribution.

Northern Europe.

###### Remarks.

Kjæranden and Søli (2020) recently reinstated the species and provided detailed figures of the male terminalia of the allied species.

##### 239. Trichonta vitta

Taxon classificationAnimaliaDipteraMycetophilidae

(Meigen, 1830)

F32B02E2-F1B1-527E-852B-9A1957148419

###### Material.

9♂♂, I-6; 1♂, SJ-4; 1♂, SJ-8; 2♂♂, K-4; 2♂♂, K-5. Total: 15♂♂.

###### Distribution in Georgia.

Imereti, Samtskhe-Javakheti, Kakheti.

###### General distribution.

Holarctic.

##### 240. Trichonta vulgaris

Taxon classificationAnimaliaDipteraMycetophilidae

Loew, 1869

83BDCAF8-3701-5FF1-88FD-E4E9205138DB

###### Material.

1♂, SZS-4 (ZFMK); 2♂♂, SJ-8; 1♂, SJ-9. Total: 4♂♂.

###### Distribution in Georgia.

Samegrelo-Zemo Svanethi, Samtskhe-Javakheti.

###### General distribution.

Holarctic.

###### Remarks.

Listed to occur in Transcaucasia without further details (Zaitzev 2003).

##### 241. Zygomyia humeralis

Taxon classificationAnimaliaDipteraMycetophilidae

(Wiedemann, 1817)

1BCCDD70-5B78-5C06-9869-EDF88C2054BE

Fig. 12J

###### Material.

1♂, SZS-3 (IZBE); 5♂♂, SZS-4 (2♂♂ ZFMK, 2♂♂ IUTG, 1♂ IZBE); 1♂, SJ-4; 2♂♂, MM-12; 1♂, MM-13; 3♂♂, MM-14. Total: 13♂♂.

###### Distribution in Georgia.

Samegrelo-Zemo Svanethi, Samtskhe-Javakheti, Mtskhetha-Mthianethi.

###### General distribution.

Europe.

##### 242. Zygomyia pseudohumeralis

Taxon classificationAnimaliaDipteraMycetophilidae

Caspers, 1980

1B78838F-81E5-5FC6-BD7F-8ADBA1E4680A

###### Material.

1♂, SJ-2 (ZFMK); 1♂, SJ-4; 1♂, SJ-7; 1♂, KK-1; 5♂♂, K-4. Total: 9♂♂.

###### Distribution in Georgia.

Samtskhe-Javakheti, Kvemo Kartli, Kakheti.

###### General distribution.

Palaearctic.

##### 243. Zygomyia semifusca

Taxon classificationAnimaliaDipteraMycetophilidae

(Meigen, 1818)

210A89BA-8F63-5F49-BDBF-C7E3973EABEE

###### Material.

1♂, SZS-4 (ZFMK); 1♂, I-6; 1♂, SJ-3; 1♂, SJ-5. Total: 4♂♂.

###### Distribution in Georgia.

Samegrelo-Zemo Svanethi, Imereti, Samtskhe-Javakheti.

###### General distribution.

Holarctic.

##### 244. Zygomyia setosa

Taxon classificationAnimaliaDipteraMycetophilidae

Barendrecht, 1938

463EE4A2-F57B-5644-90FE-C77C2EBB1AE0

###### Material.

1♂, K-4. Total: 1♂.

###### Distribution in Georgia.

Kakheti.

###### General distribution.

Europe.

###### Remarks.

A very rare species with a scattered distribution in Europe: recorded from the Netherlands, Germany and Switzerland (Chandler 2013). There is an unpublished record also from Estonia (personal observation).

##### 245. Zygomyia valida

Taxon classificationAnimaliaDipteraMycetophilidae

Winnertz 1863

168B76C8-5058-5C37-ADDA-15117E805C9F

###### Material.

10♂♂, SZS-4 (4♂♂ ZFMK, 3♂♂ IUTG, 3♂♂ IZBE). Total: 10♂♂.

###### Distribution in Georgia.

Samegrelo-Zemo Svanethi.

###### General distribution.

Palaearctic.

##### 246. Zygomyia vara

Taxon classificationAnimaliaDipteraMycetophilidae

(Staeger, 1840)

5A1DC03A-FFC5-5FD2-BF50-FC164B06B610

###### Material.

1♂, SZS-2; 1♂, SZS-4 (ZFMK); 1♂, A-1; 2♂♂, SJ-9. Total: 5♂♂.

###### Distribution in Georgia.

Samegrelo-Zemo Svanethi, Adjara, Samtskhe-Javakheti.

###### General distribution.

Holarctic.

###### Remarks.

Listed to occur in Transcaucasia without further details (Zaitzev 2003).

## Discussion

**Figure 13.**
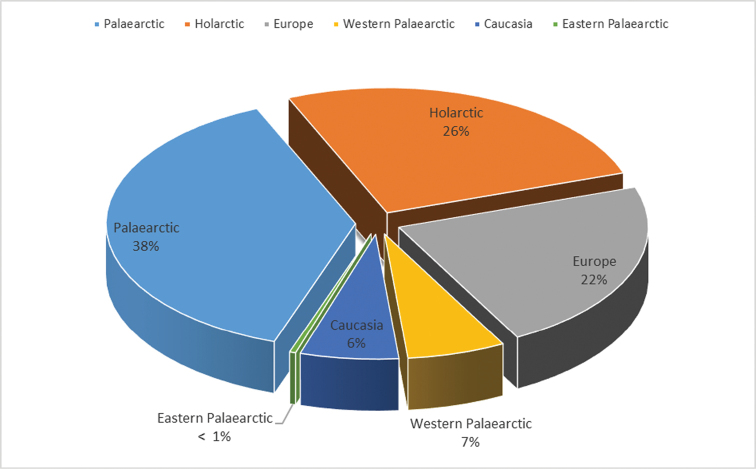
Grouping of the recorded Georgian fungus gnat species in accordance with their known distribution.

This is the first attempt to provide a synoptic list of Sciaroidea species of the Transcaucasian region. However, the recorded 246 species (245 from original study + one from literature data) of fungus gnats are the result of a preliminary survey, while further sweeping studies will probably increase that number considerably (see also below). As expected, the majority of the recorded species are widely distributed in the Palaearctic or Holarctic regions (38% and 26% of the recorded species, respectively), while 22% of species are restricted to Europe and 7% to the Western Palaearctic (Fig. 13). In addition, one species was so far known only from the Eastern Palaearctic and 17 species (11 described and 6 undescribed) are classified (tentatively) as Caucasian endemics. These proportions can change as fungus gnats are rather poorly known in several Palaearctic regions including the East Palaearctic, Asia Minor, Central Asia, as well as other regions in Caucasia.

**Figure 14.**
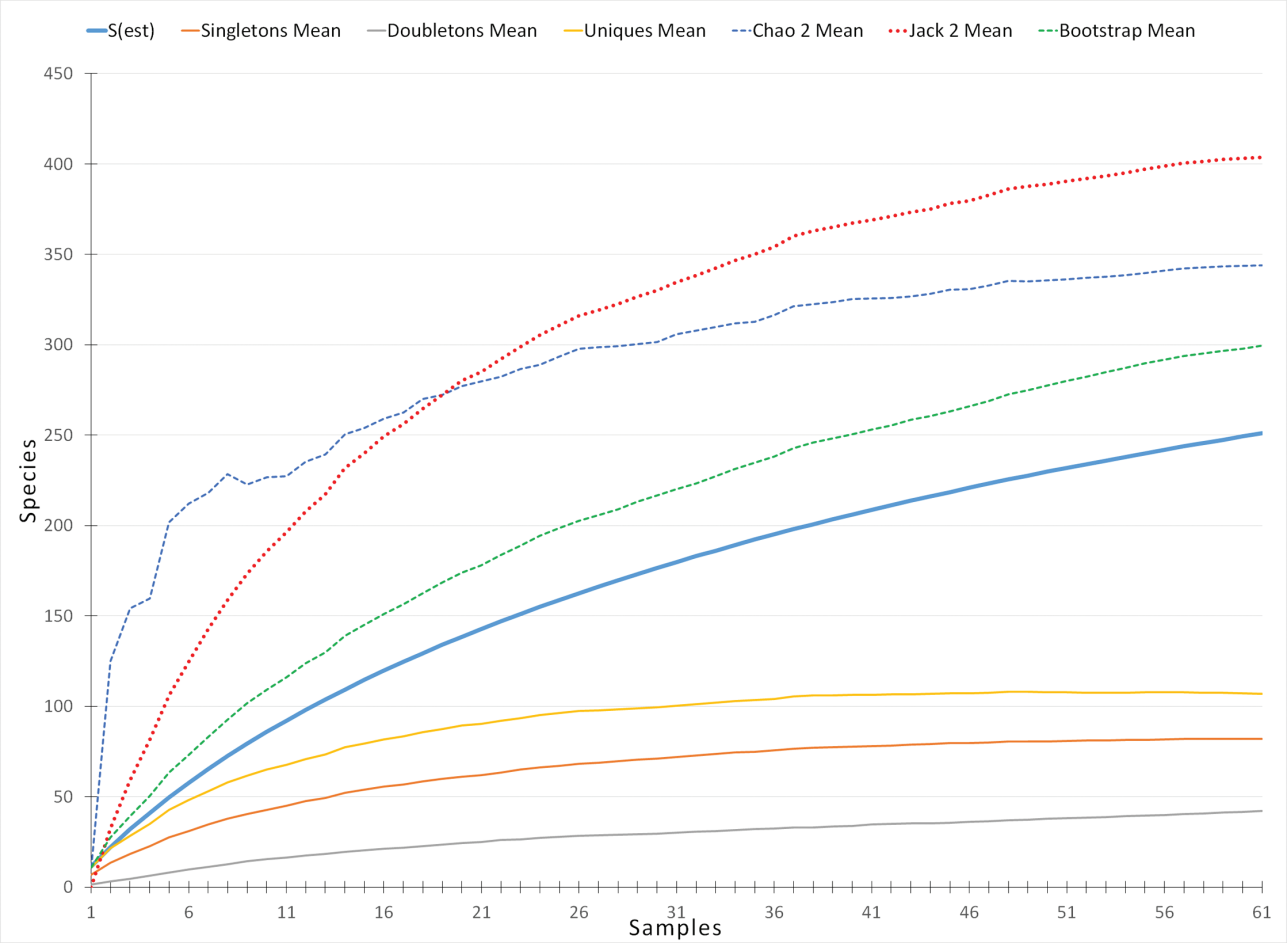
Species accumulation curves (EstimateS, Vesrion 9.1.0.). Three nonparametric estimators (Chao 2, Jackknife 2 and Bootstrap) of total species richness are calculated. S(est) is the cumulative number of species observed.

The estimated species richness is the highest when calculated using Jackknife-2 nonparametric estimator (404 species, Fig. 14). This method has been discussed as possibly overestimating the true richness (e.g. Poulin 1998). On the other hand, Smith and van Belle (1984) showed that both Jackknife and Bootstrap estimators underestimate the actual number of species if there is a large number of rare species considered and number of samples is low. That can also be the case in the current data as the number of recorded singletons and doubletons is exceptionally high (82 and 42 species, respectively) and the number of species recorded from one sample only (= unique species) constitutes 43% of the observed diversity (107 out of the 251). Within the listed species, only nine were recorded from more than ten samples and 31 species from 5–10 samples. To compare, relatively well studied countries of similar size in Central and Northern Europe (e.g. Czech Republic, Slovak Republic, Estonia) have roughly 600 fungus gnat species recorded (Ševčík and Košel 2009, Ševčík and Kurina 2011a, b, *pers. observation*). Taking into account the mountainous landscape, high diversity of habitats, microclimates in Georgia and that several regions were not covered by sampling of the current study (see Fig. 1), it can be presumed that the observed 245 species (+ one based on the literature data) do not constitute more than half of the actual diversity, probably less.

Surprisingly, the most abundant species was *Synapha fasciata* (769 specimens from 19 samples) followed by *Orfelia georgica* (175 specimens from 14 samples). In the European boreal and temperate regions, the most abundant species belong frequently to the subfamily Mycetophilinae and/or to the genera *Boletina* Staeger and *Mycomya* Rondani. Several of the recorded species considerably increase the knowledge of their distribution, the most remarkable of them include: *Neoempheria brevilineata* (earlier from Japan only), *Clastobasis loici* (earlier from Europe and Japan), *Lusitanoneura chandleri* (earlier from the Mediterranean Islands), *Zygomyia setosa* (earlier with scattered distribution in Europe), *Manota unifurcata* (earlier from Europe only).

From the material underlining this study, four new species have been described earlier (Kurina and Jürgenstein 2013; Kurina 2018; Ševčík et al. 2020), three new species are described above and six putatively new species are left to be described in the future due to insufficiency of the available material or its quality. More exhaustive sampling will naturally yield a number of new taxa to be described in the future.
